# Impact of age on clinical outcomes among patients with hepatocellular carcinoma: A systematic review and meta-analysis

**DOI:** 10.1016/j.jhepr.2025.101368

**Published:** 2025-02-26

**Authors:** Olgert Bardhi, Darine Daher, Mausam Patel, Karim Seif El-Dahan, Nicole E. Rich, Sukul Mitta, Neehar D. Parikh, Anjana Pillai, Laura M. Kulik, Ju Dong Yang, Anand V. Kulkarni, Purva Gopal, Amit G. Singal

**Affiliations:** 1 Department of Internal Medicine, University of Texas Southwestern Medical Center, Dallas, TX, USA; 2 Department of Internal Medicine, University of Michigan, Ann Arbor, MI, USA; 3 Department of Internal Medicine, University of Chicago, Chicago, IL, USA; 4 Department of Internal Medicine, Northwestern University, Evanston, IL, USA; 5 Department of Internal Medicine, Cedars-Sinai Medical Center, Los Angeles, CA, USA; 6 Department of Hepatology and Liver Transplantation, AIG Hospitals, Hyderabad, India

**Keywords:** Liver cancer, Elderly, Disparities, Prognosis, Treatment

## Abstract

**Background & Aims:**

Older adults have lower treatment eligibility and worse survival across cancer types; however, the association between age and outcomes in patients with hepatocellular carcinoma (HCC) has not been well characterized.

**Methods:**

We performed a search of the PubMed, Ovid MEDLINE, and EMBASE databases from January 2000 to July 2022 to identify studies reporting tumor stage, curative treatment, and overall survival among patients with HCC, stratified by age. Using the DerSimonian and Laird method for a random-effects model, we calculated pooled risk ratios (RRs) for curative treatment receipt and hazard ratios (HRs) for overall survival among younger and older patients (per age thresholds in each study).

**Results:**

We identified 103 studies (n = 154,152 patients) that reported outcomes in younger *vs*. older patients with HCC. Younger patients were more likely to undergo curative treatment (RR 1.48, 95% CI 1.24–1.77; I^2^ = 99%), although few studies reported treatment among those with early-stage HCC. Younger patients had better survival than older patients (HR 0.87, 95% CI 0.83–0.92; I^2^ = 89%), which was consistent in subgroups using age thresholds of <70 years (HR 0.94, 95% CI 0.89–0.99; I^2^ = 78%) and <75 years (HR 0.83, 95% CI 0.70–0.98; I^2^ = 79%). Younger patients also had better survival in studies of patients with early-stage HCC (HR 0.78, 95% CI 0.65–0.94; I^2^ = 60%) and those undergoing curative therapy (HR 0.87, 95% CI 0.77–0.98; I^2^ = 87%).

**Conclusions:**

Older patients with HCC are less likely to receive curative treatment and have worse survival than their younger counterparts. Studies to identify factors associated with worse prognosis can inform intervention targets.

**Impact and implications:**

Older adults have worse survival across cancer types, although there are discordant data about the association between age and clinical outcomes in patients with hepatocellular carcinoma (HCC). Lower curative treatment receipt among older patients, despite similar early-stage presentation compared with younger patients, requires future studies to identify mediators that can inform intervention strategies that can increase curative treatment use. Worse survival observed among older patients appears to be primarily driven by non-liver-related mortality; however, few studies distinguish between liver and non-liver mortality. A better understanding of the prognostic value of comorbidity burden, in addition to age, can inform clinical decisions about stopping rules for HCC surveillance as well as the potential for HCC overdiagnosis and overtreatment.

## Highlights

• Early-stage HCC presentation did not significantly differ between younger and older patients.
• However, younger patients are more likely to receive curative treatments than older patients.
• Younger patients demonstrate a lower risk of mortality than older patients.
• These findings emphasize the need for tailored approaches in older populations to reduce survival disparities.

## Graphical abstract

**Figure:**
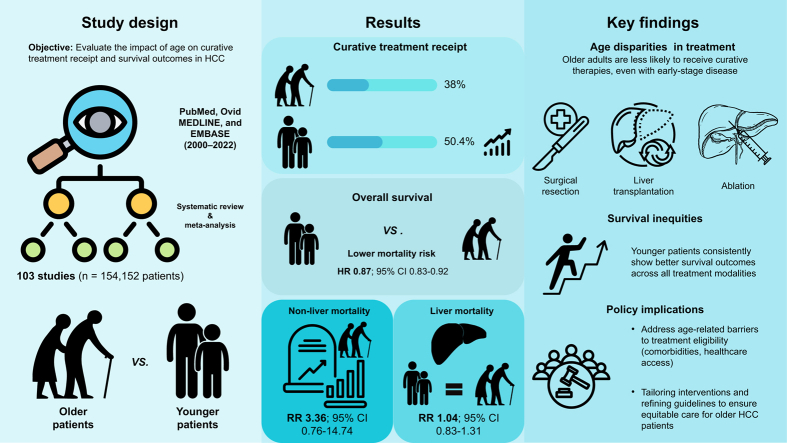

## Introduction

Hepatocellular carcinoma (HCC) is a leading cause of cancer-related mortality in patients with cirrhosis, with a 5-year survival below 25%.^1^^,^^2^ The strongest drivers of HCC prognosis are tumor stage at presentation and receipt of curative therapy.^3^ Patients with early-stage HCC who undergo surgical procedures achieve a 5-year survival rate surpassing 70%.^4^ This survival is in stark contrast to those with more advanced tumors receiving palliative therapies, who experience a 5-year survival below 30%.^4^

The age-dependent nature of HCC risk is well established, with HCC incidence peaking after age 60 years in Western countries.^3^ The increasing life expectancy of the population, including patients with cirrhosis, is projected to result in increased numbers of older individuals diagnosed with HCC.^4^ Consequently, understanding the presentation and management of HCC in this patient population is paramount.

Effective management of older patients with cancer poses substantial challenges primarily because of heightened susceptibility to concurrent comorbid conditions, which can hinder access to appropriate treatments.^5^ Studies from colorectal and lung cancers suggest that older patients are often less likely to receive treatment compared with younger counterparts with similar tumor burden.^6^^,^^7^ Similarly, older patients with HCC may be less likely to receive treatment than younger counterparts. Furthermore, data on the efficacy and tolerability of cancer treatments in elderly patients is often limited, as this group is underrepresented in clinical trials.^8^

Compared with other cancers in which age and comorbidity have been incorporated into “stopping rules” for cancer screening, HCC surveillance is recommended in patients with cirrhosis or chronic HBV infection without established stopping rules based on age.^9^^,^^10^ However, a recent modeling study suggested that HCC surveillance may not be cost-effective in patients older than 70 years after hepatitis C cure.^11^ This recommendation was partly based on competing risk of mortality and presumed lower eligibility for curative treatments.

Understanding outcomes among older adults is important to guide recommendations for optimizing HCC management strategies. However, no studies have comprehensively reviewed age-based differences in treatment receipt and clinical outcomes in patients with HCC. Therefore, we conducted a systematic review and meta-analysis to better understand differences in presentation, treatment receipt, and overall survival between younger and older patients with HCC.

## Materials and methods

This systematic review was conducted in accordance with the Preferred Reporting Items for Systematic Reviews and Meta-Analyses (PRISMA) reporting guidelines.^12^

### Search strategy

We performed an electronic-based search of the PubMed, Ovid MEDLINE, and EMBASE databases to identify all relevant articles and abstracts evaluating studies reporting tumor stage, curative treatment receipt, and/or overall survival among patients with HCC, stratified by age (younger *vs*. older) published between January 2000 and July 2022. The search terms included the following: (liver ca∗ OR HCC OR hepatocellular ca∗) AND (elderly OR old∗ OR young∗). Manual searches of reference lists were also conducted to identify citations that may have been missed by the electronic-based search. Only English articles were considered for further analysis.

### Study selection and inclusion/exclusion criteria

Following removal of duplicate citations and the application of inclusion/exclusion criteria, one investigator (OB) screened titles, abstracts, and full texts of the remaining citations to compile a list of potentially relevant articles. A second investigator independently reviewed full texts of included articles for eligibility confirmation and data abstraction, with a third investigator available to resolve disagreements. To mitigate the possibility of missing studies with age-stratified outcomes as a secondary analysis, we used lenient title and abstract screening criteria, with a greater number of studies undergoing full-text review.

Eligible studies encompassed randomized controlled trials and observational studies involving HCC patients aged 18 years or older. We excluded studies that (1) lacked a comparison between younger and older HCC patients, (2) did not include data on treatment receipt or overall survival, (3) were not available in the English language, (4) involved nonhuman data, or (5) were comprised of case reports, review articles, or editorials. In cases of duplicate publications featuring the same patient cohort, the study with the most complete data or most recent was included.

### Data extraction and quality assessment

Two investigators (OB and DD) independently extracted data from eligible full texts, with a third investigator (AGS) available to resolve discrepancies. Data elements included author, publication year, country, study design, number of patients, age thresholds defining younger *vs*. older, follow-up duration, proportion of early-stage HCC, type of HCC treatments, and survival estimates. Study quality and bias risk were evaluated using a modified National Institutes of Health Study Quality Assessment Tool.^13^

#### Exposure of interest

The definition of “younger” *vs*. “older” was determined based on the age threshold specified in each study. In cases where studies reported multiple age thresholds, we used thresholds of either 65 or 70 years for the primary analyses, as these were most commonly reported across studies. Sensitivity analyses were conducted for other reported age thresholds.

#### Outcome measures

Clinical outcomes of interest included early-stage HCC, curative treatment receipt, and overall survival. For early-stage HCC, we captured the proportion of patients detected at an early stage using the definition of early-stage HCC in each study. If multiple definitions were provided, we preferentially used the Milan criteria or Barcelona Clinic Liver Cancer (BCLC) stage.^14^^,^^15^ All HCC treatments were recorded, including liver transplantation, surgical resection, local ablation therapy, transarterial therapy (transarterial chemoembolization or transarterial radioembolization), stereotactic body radiation therapy, and systemic therapy; curative treatments was defined as liver transplantation, surgical resection, or local ablation.

### Statistical analysis

For each study, we computed the proportions of early-stage HCC and receipt of curative therapy in younger *vs*. older patients. We then calculated pooled risk ratios (RRs) accompanied by 95% CIs for both outcomes using the DerSimonian and Laird method for a random-effects model. We used the χ^2^ test of heterogeneity and consistency index (I^2^) to quantitatively determine the extent of heterogeneity between studies. I^2^ values >75% indicate a high level of heterogeneity, whereas I^2^ values between 50% and 75% indicate moderate heterogeneity. We then conducted subgroup analyses to investigate potential sources of heterogeneity. These analyses were preplanned for the following categories: (1) geographic region (Asia *vs*. Europe *vs*. USA *vs*. other), (2) publication year (before 2005, 2005–2009, 2010 –2014, and 2015–2022), (3) type of curative treatment, and (4) age threshold. To assess potential publication bias, we used both visual examination of funnel plots and statistical analysis using Egger’s test.^16^

To compare early-stage HCC presentation and treatment receipt between younger and older patients, we calculated a pooled RR. To compare mortality between younger and older patients, we calculated pooled hazard ratios (HRs). For studies that reported HR for survival without a corresponding 95% CI, we computed the 95% CI using the effect estimate and *p* value. For studies reporting more than two HR values, we used the reference age group as the age threshold and conducted pooled analyses of HR values among older patients. Finally, for studies that did not report HR but included 5-year survival data, we calculated the odds ratio (OR) for 5-year survival between younger and older patients. All analyses were conducted using R Studio version 4.2.1 (R Foundation for Statistical Analysis, Vienna, Austria).

## Results

### Study characteristics

Our literature search identified 14,265 relevant titles published from January 2000 to July 2022. Following screening of titles, 1009 abstracts were reviewed. After a full-text review, 103 studies met the inclusion criteria (Fig. S1); characteristics of these studies are detailed in Tables S1 and S2. Among 154,152 total patients, 101,597 (65.9%) were categorized as “younger” and 52,555 (34.1%) as “older” based on study-reported thresholds. Age thresholds varied across studies, with the most common being 70 years (n = 39) and 75 years (n = 25).

The studies were geographically diverse with 32 conducted in Japan, 18 in China, and 20 in other Asian countries; 15 studies were conducted in Europe, 13 in the US, and the remaining studies elsewhere. Seven studies were published before 2005, 14 between 2005 and 2009, 36 studies 2010 and 2014, and 46 after 2014. There were 26 “all-comer” studies that included patients regardless of tumor stage and treatment receipt, whereas the other studies included focused cohorts by tumor stage or treatment type. Among the 50,931 patients in the “all-comer” studies with receipt of any curative treatment, 32,370 were categorized as “younger” and 18,561 as “older.”

### Early-stage presentation

Among 21 studies examining tumor stage at presentation (n = 17,813 patients), no difference in early-stage HCC was observed between younger and older patients (RR 1.00, 95% CI 0.94–1.07; I^2^ = 35%) (Fig. S2). Early-stage HCC was detected in 36.3% of younger patients *vs*. 35.0% of older patients. Similarly, among the subset of 13 studies that defined “early stage” using BCLC staging or Milan criteria, there was no significant difference in early-stage presentation between younger and older patients (RR 1.02, 95% CI 0.96–1.08; I^2^ = 15%). In subgroup analyses, no significant differences were noted in early-stage presentation using age thresholds of 65, 70, and 75 years. Results remained consistent across other subgroups, including geographic location and study period, although there were greater improvements in early-stage detection over time among younger patients than among older patients (Table S3).

### Treatment receipt

**Fig. 1:**
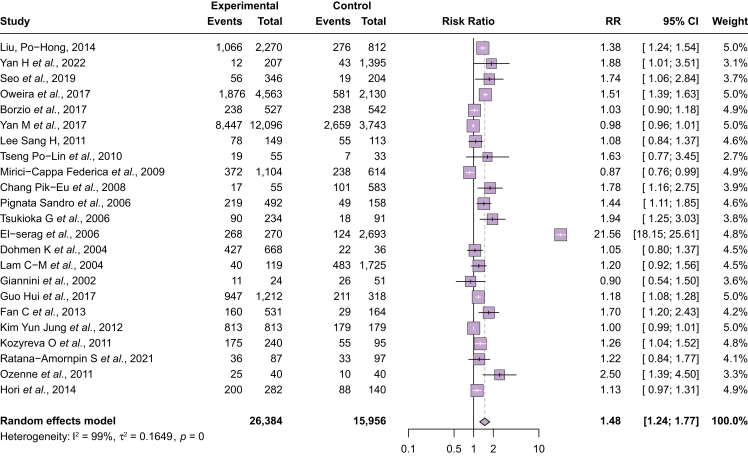
Receipt of any curative treatment between younger and older patients. Younger patients were more likely to receive curative treatment than older patients (RR 1.48, 95% CI 1.24–1.77). RR, risk ratio.

Among 23 studies reporting curative treatment receipt (n = 42,340 patients), younger patients were more likely to receive curative treatment than older patients (RR 1.48, 95% CI 1.24–1.77) (Fig. 1). Younger patients had curative treatment receipt in 50.4% (40.0–60.6%) of cases compared with older patients at 38.0% (27.6–48.4%) (Table S3). Pooled results were limited by high heterogeneity (I^2^ = 99%), which was explored in sensitivity and subgroup analyses. Notable outliers on forest plot inspection were the studies by El-Serag *et al.*^17^ and Ozenne *et al.*^18^ When these studies were excluded, pooled results remained similar (RR 1.22, 95% CI 1.10–1.37) although heterogeneity persisted (I^2^ = 97.4%). When considering various age thresholds defining “older” *vs*. “younger,”, there was no difference in curative treatment receipt at thresholds of 65 or 70 years; however, significant differences were noted at a threshold of 75 years (RR 1.37, 95% CI 1.07–1.75; I^2^ = 80%). Across geographic regions, significant differences were not observed in studies from Asia or Europe, although younger patients were more likely to undergo curative treatment than older patients among studies from the USA (RR 2.51, 95% CI 1.02–6.18; I^2^ = 100%). Although there was no significant difference in curative treatment receipt between younger and older patients among studies published before 2015, curative treatment was more likely in younger patients among studies published in 2015 or later (RR 1.25, 95% CI 1.02–1.52; I^2^ = 96%) (Table S3). Studies attributed differences in curative treatment receipt to several factors including liver dysfunction, performance status, and comorbidity burden (Table S4).

Only three studies compared curative treatment receipt between younger and older patients with early-stage HCC. Guo *et al.*^19^ found that older and younger patients with BCLC stage 0-A HCC were equally likely to receive curative therapy (65% *vs*. 65%). Liu *et al.*^20^ found that elderly patients were more frequently treated with local ablation than surgical resection compared with younger patients owing to preoperative risk assessment. Conversely, Oweira *et al.*^21^ evaluated 6693 patients ≥70 years with early-stage (T1/T2) HCC in the SEER registry and found those aged 70–80 years were more likely to receive surgical or local ablative therapy than those aged >80 years (41.1% *vs*. 27.3%).

### Overall survival

A total of 50 studies (n = 123,293) examined HCC survival with outcomes stratified by age. Compared with older patients, younger patients had better survival (HR 0.87, 95% CI 0.83–0.92), although there was a high level of heterogeneity (I^2^ = 89%) (Fig. S3). Absolute survival estimates among younger and older patients are described in Table S5**.** When outliers on visual inspection of the forest plots were excluded, younger patients continued to have better survival (HR 0.87, 95% CI 0.83–0.92), and heterogeneity remained high (I^2^ = 89%). When considering various age thresholds to define “younger” *vs*. “older” patients, there were significant differences in survival at thresholds of 70 years (HR 0.94, 95% CI 0.89–0.99; I^2^ = 78%) and 75 years (HR 0.83, 95% CI 0.70–0.98; I^2^ = 79%) but not at lower thresholds of 60 or 65 years. Improved survival in young patients was reported in studies across geographic locations including the USA (HR 0.88, 95% CI 0.78–0.99; I^2^ = 94%), Europe (HR 0.80, 95% CI 0.66–0.98; I^2^ = 88%), and Asia (HR 0.91, 0.84–0.99; I^2^ = 78%). There was no difference in survival among studies published between 2005 and 2009 (HR 1.02, 95% CI 0.79–1.31; I^2^ = 85%); however, younger patients had better survival in studies published between 2010 and 2014 (HR 0.89, 95% CI 0.84–0.95; I^2^ = 83%) and in 2015 or later (HR 0.88, 95% CI 0.81–0.96; I^2^ = 80%).

**Fig. 2:**
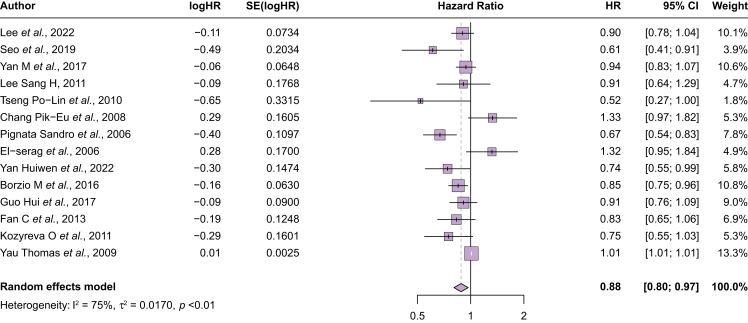
Overall survival between younger and older patients. Younger patients had overall better survival compared with older patients (HR 0.88, 95% CI 0.80–0.97). HR, hazard ratio.

Among the 14 all-comer studies (n = 33,699) with sufficient data to calculate a pooled estimate, younger patients had better survival (HR 0.88, 95% CI 0.80–0.97; I^2^ = 75%) (Fig. 2). When outliers on visual inspection of the forest plots (three with lower hazards and two with higher hazards of mortality) were excluded, younger patients continued to have better survival (HR 0.90, 95% CI 0.83–0.98), and heterogeneity was reduced, albeit still moderate (I^2^ = 66%). Across age thresholds, a significant difference in survival was observed at a threshold of 70 years (HR 0.81, 95% CI 0.69–0.96; I^2^ = 84%) but not at age 65 years. Survival did not significantly differ by age among studies conducted in Asia (HR 0.90, 95% CI 0.80–1.01; I^2^ = 67%) or the USA (HR 0.97, 95% CI 0.75–1.24; I^2^ = 66%); however, improved survival in younger patients was reported in the two studies from Europe (HR 0.77, 95% CI 0.61–0.97; I^2^ = 72%). Finally, survival did not significantly differ between younger and older patients among studies published between 2005 and 2009 (HR 1.02, 95% CI 0.79–1.31; I^2^ = 85%), although differences were observed in studies published between 2010 and 2014 (HR 0.80, 95% CI 0.68–0.94; I^2^ = 0%) and those published in 2015 or later (HR 0.87, 95% CI 0.81–0.94; I^2^ = 19%).

Few studies distinguished liver *vs*. non-liver mortality between younger and older patients, with most simply reporting the proportion of liver-related *vs*. non-liver-related deaths in both subgroups (Table S6). Two studies reported outcomes among patients undergoing a broader range of treatments. Kozyreva *et al.*^22^ provided liver-related morality rates for younger and older patients, including median survivals of 24.5 *vs*. 27.8 months and 1-year survival of 66.7% and 66.5%, respectively; however, higher non-liver mortality was observed among older individuals. Conversely, Lee *et al.*^23^ found similar liver and non-liver mortality between younger and older individuals. Other studies reported liver and non-liver mortality among specific subgroups of patients, such as those undergoing surgical resection or liver transplantation. Most studies found higher non-liver mortality among older individuals; however, studies were discordant about differential liver-related mortality, with some reporting higher liver mortality among older individuals and others reporting similar mortality.

Of the all-comer studies, only three (n = 922) reported data comparing liver-related *vs*. non-liver-related mortality in younger and older patients. Liver-related mortality did not significantly differ between the two age groups (RR 1.04, 95% CI 0.83–1.31; I^2^ = 64%), with liver-related mortality in 52% (95% CI 48.3–55.7%) of older patients and 50% (95% CI 41.9–58.2%) of younger patients (Fig. S5). Conversely, non–liver-related deaths were higher among older patients (25.5%, 95% CI 3.5–54.6%) than among younger patients (5.0%, 95% CI 1.6–8.3%), with a relative risk of 3.36 (95% CI 0.76–14,7; I^2^ = 90%) (Fig. S5).

### Overall survival among patients with early-stage HCC

**Fig. 3:**
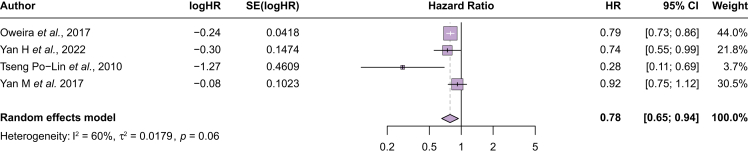
Overall survival between younger and older patients with early-stage HCC. Younger patients with early-stage HCC had overall better survival compared with older patients (HR 0.78, 95% CI 0.65–0.94). HCC, hepatocellular carcinoma; HR, hazard ratio.

Nine studies compared survival among patients with early-stage HCC (Table S7). In four studies with data to calculate a pooled estimate, younger patients had better survival than older patients (HR 0.78, 95% CI 0.65–0.94; I^2^ = 60%) (Fig. 3). When Tseng *et al.*^24^ was excluded as an outlier study, younger patients continued to have better survival (HR 0.81, 95% CI 0.74–0.88; I^2^ = 10%) and heterogeneity was minimal (I^2^ = 10%). Of the five studies that compared survival outcomes among older and younger patients with early-stage HCC but did not report HRs (Table 1), three found that younger patients had significantly better survival compared with older patients and two found numerically higher but not significantly improved survival in younger patients. The study by Tsukioka *et al.*^26^ found that younger patients with TNM stage I/II had a 5-year survival of ∼38% compared with 0% for older patients (≥80 years of age). Borzio *et al.*^25^ reported that younger patients with BCLC 0-A had a median survival of >60 months compared with 44 months for older patients (>70 years of age). Guo *et al.* found that younger patients had higher overall survival (37 *vs*. 44 months; *p* >0.05).

**Table 1:** Studies reporting overall survival in patients with early-stage HCC.

Study, year	Country	Age cut-off (years)	Number of patients	Staging system	Overall survival in younger *vs*. older, months (95% CI)
Lee 2011^23^	South Korea	<65 ≥65	<65, n = 149 ≥65, n = 113	BCLC A TNM I	57.3 (52.2–62.4) *vs*. 53.6 (46.3–60.8), *p* = 0.26 56.9 (48.6–65.1) *vs.* 45.2 (36.5–54.0), *p* = 0.09
Borzio 2016^25^	Italy	≤70 >70	≤70, n = 527 >70, n = 542	BCLC 0-A	>60.0 *vs*. 44.0, *p* <0.001
Guo 2017^19^	China	<65 ≥65	<65, n = 1,212 ≥65, n = 318	BCLC 0-A	44.0 *vs*. 37.0, *p* = 0.05
Tsukioka 2006^26^	Japan	≥80 50–60	≥80, n = 91 50–60, n = 34	TNM I TNM II	120.0 *vs*. 50.0∗, *p* = 0.005
Kim 2012^27^	South Korea	<70 ≥70	<70, n = 813 ≥70, n = 179	BCLC 0 BCLC A TNM I	76.4 (68.9–83.8) *vs*. 63.0 (45.8–80.3), *p* = 0.36 68.2 (60.6–75.8) *vs*. 72.5 (56.6–88.4), *p* = 0.73 68.6 (58.9–78.2) *vs*. 70.9 (53.1–88.6), *p* = 0.55

∗ Derived from Kaplan Meier curves in Fig. 1 of article. BCLC, Barcelona Clinic Liver Cancer; TNM, tumor node metastasis.

### Overall survival among patients after curative treatment

**Fig. 4:**
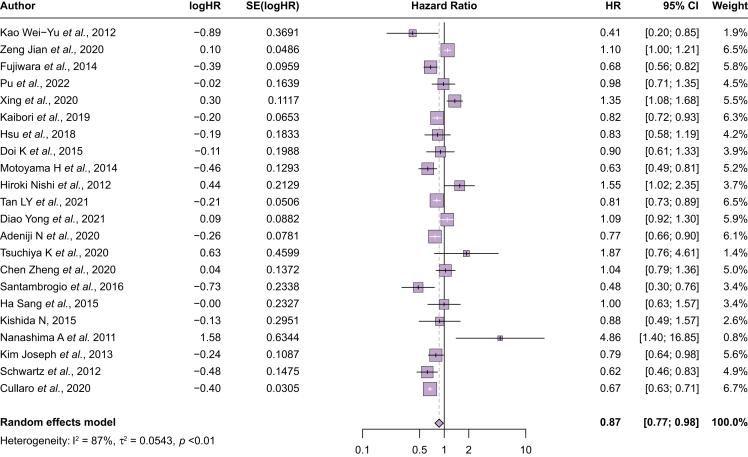
Overall survival between younger vs. older patients who underwent curative treatment. Younger patients who underwent curative treatment had overall better survival compared with older patients (HR 0.87, 95% CI 0.77–0.98). HR, hazard ratio.

Finally, 63 studies compared survival among those who underwent curative therapy (49 with surgical resection, seven orthotopic liver transplantation, and seven radiofrequency ablation) (Table S2). In the 22 studies with data to calculate a pooled HR, younger patients had better survival than older patients (HR 0.87, 95% CI 0.77–0.98; I^2^ = 87%) (Fig. 4). After the exclusion of outliers visualized on the forest plot (two with lower hazards and three with higher hazards), younger patients continued to have better survival (HR 0.86, 95% CI 0.76–0.96; I^2^ = 87%). There was no difference in survival between younger and older patients who underwent surgical resection or local ablative therapies; however, younger patients who underwent liver transplantation had improved survival compared with older patients (HR 0.71, 95% CI 0.64–0.78; I^2^ = 39%). Differences in survival were greater in magnitude among studies before 2010 but did not reach statistical significance among studies between 2010 and 2014 (HR 0.79, 95% CI 0.60–1.03; I^2^ = 78%) or after 2015 (HR 0.91, 95% CI 0.79–1.04; I^2^ = 89%). Across age thresholds, a significant difference in survival was observed at a cuff-off of 65 years (HR 0.75, 95% CI 0.61–0.92; I^2^ = 78%), but not at higher thresholds of 70 or 75 years.

Sixty-two studies provided data on 5-year survival, among which younger patients had higher 5-year survival (OR 1.21, 95% CI 1.10–1.32; I^2^ = 75%) (Fig. S4). Subgroup analysis by type of curative treatment showed improved survival among younger patients receiving surgical resection (OR 1.15, 95% CI 1.03–1.28; I^2^ = 67) and local ablative therapies (OR 1.48, 95% CI 1.15–1.91; I^2^ = 54%), but differences did not reach significance among those undergoing liver transplantation (OR 1.31, 95% CI 0.999–1.71; I^2^ = 92%).

### Quality assessment

We found no evidence of publication bias for early-stage HCC presentation (*p* = 0.05), although there was potential publication bias for studies examining curative treatment (*p* = 0.04) and overall survival (*p* = 0.008). Examination of funnel plots suggested a dearth of studies with smaller sample sizes, particularly small negative studies on curative treatment and overall survival differences.

Regarding quality assessment, most studies had clearly defined eligibility criteria and objective outcomes of interest. The most notable limitation of the existent literature was a risk of residual confounding, with only 24 studies matching for important variables including tumor burden, degree of liver dysfunction, and comorbidity. There were an additional 67 studies that adjusted for some but not all confounders. Notably, many studies failed to differentiate liver-related *vs*. non-liver-related mortality. Other limitations for most studies were loss to follow-up exceeding 20% at 5 years or loss to follow-up not being reported.

## Discussion

Given the increasing number of HCC cases diagnosed in older adults and an aging at-risk population, it is important to understand age-related differences in HCC treatment patterns and prognosis. Despite a similar proportion of younger and older patients having tumors diagnosed at an early stage, we found that younger patients were significantly more likely to undergo curative treatment and exhibit better overall survival. These differences in survival persisted when examining the subset of patients detected at an early stage and the subset that received curative treatment. Age thresholds to define “older” *vs*. “younger” were inconsistent across studies; however, differences in curative therapy and survival appeared greatest in patients ≥75 years. Although there were no significant differences in curative treatment receipt, early-stage HCC detection, or overall survival between younger and older patients during earlier study periods, these differences became more pronounced over time. There appeared to be greater improvements in early-stage presentation and overall survival over time among younger than older patients.

We found no significant differences in early-stage detection between younger and older individuals. Although our included studies did not provide data on HCC surveillance receipt, prior meta-analyses found no significant differences by age.^28^ Despite similar proportions of older adults being diagnosed with early-stage HCC, they are less likely to receive curative treatment. The reasons behind this disparity are complex and may be attributed to many possible clinical factors including differences in liver dysfunction, performance status, or comorbidity burden.^5^^,^^29^ Alternatively, this disparity may also relate to differences in patient preferences such as decreased willingness to proceed with invasive surgical therapies, differences in social support to engage in healthcare decisions, differences in access (*e.g.* ability to drive to appointments), or implicit physician bias. We found that the greatest disparity in curative treatment receipt was among patients aged ≥75 years, which may be driven by differential access to surgical procedures.^30^^,^^31^ Although there is no universally accepted age threshold for liver transplantation, older patients may derive fewer life-years gained.^32^^,^^33^ Interestingly, we found no significant differences in treatment receipt at lower age thresholds, specifically >65 years, suggesting acceptance of surgical therapies by patients and providers for patients aged 65–75 years.

Finally, we found that older patients had significantly worse survival than younger patients overall, likely in part related to lower rates of curative treatment receipt. However, this disparity persisted among the subgroups of patients with early-stage HCC and those who underwent curative treatment. Once again, the factors driving this disparity are likely multifactorial, with potential factors including age-related differences in liver dysfunction, prevalence of comorbid conditions, and overall performance status. Interestingly, the greatest difference was observed in the subset of patients who underwent liver transplantation, despite generally stringent selection criteria aimed at identifying optimal candidates for transplant. Although there are different HCC subtypes and variation in tumor biology, prior studies have not reported more aggressive tumor biology in older patients compared with younger patients.^34^^,^^35^ Therefore, this survival difference may instead be related to a higher competing risk of non-liver-related mortality in older patients.

Further studies are needed to characterize reasons driving differences in curative treatment to see if this disparity can be mitigated. Conversely, the benefits of early-stage HCC detection may be mitigated in older patients, and these data would inform stopping rules for HCC surveillance programs. Stopping rules are helpful to maximize the overall value of surveillance programs, as older patients may be at greatest risk of surveillance harms but derive fewer benefits.[36], [37], [38], [39], [40] Importantly, future studies will need to examine the intersection between age and other factors such as comorbidity burden and liver dysfunction. For example, surveillance is likely to be of greater benefit in an otherwise healthy 75-year-old patient with Child– Pugh A cirrhosis than in a 65-year-old patient with Child–Pugh B cirrhosis and significant comorbidities (*e.g.* congestive heart failure or renal failure). These concepts have been incorporated into other cancer screening programs such as colorectal cancer screening and can similarly be considered for HCC surveillance.^41^ The AASLD recommends consideration of comorbidity, performance status, and patient preferences when assessing the value of HCC surveillance in individuals, but further data are needed to codify how to incorporate these factors into decisions.

Our results should be interpreted considering the study’s limitations. First, our meta-analysis was conducted at the study level, and we did not have patient-level data for additional analyses to explore observed age disparities. Specifically, this precluded our ability to examine important confounders, including liver dysfunction and comorbidity. Second, several of our pooled analyses had moderate to severe heterogeneity, which we tried to address through subgroup analyses; however, heterogeneity could not be fully resolved in all cases. The heterogeneity is evident with variations in age thresholds, study populations, and definitions of early-stage HCC across studies. Third, there was a suggestion of publication bias with potential under-reporting of small negative studies. Fourth, some studies with relevant age-stratified outcomes may have been missed by our literature search, particularly if not the primary aim of the study; however, we attempted to mitigate this possibility by using lenient title and abstract screening criteria, with a greater number of studies undergoing full-text review. Fifth, studies evaluating survival after curative treatments may be prone to differential selection bias among younger and older patients, and it is unclear if the same findings would hold among broader populations eligible for curative treatment. Finally, the interpretation of the pooled results in our meta-analysis is limited by the quality of the included studies. Many studies had a risk of residual confounding and failed to report loss to follow-up for survival analysis. Further, most studies failed to account for intersectionality between other known differences in HCC treatment and prognosis, including race/ethnicity, socioeconomic status, and rural–urban geography.[42], [43], [44], [45] Many studies also did not differentiate liver *vs*. non-liver mortality despite the expected higher non-liver mortality among older adults. These gaps in analyses and reporting should be addressed in future studies examining potential differences in outcomes by age.

### Conclusions

There are consistent and persistent age differences in HCC treatment and prognosis, with younger patients more likely to receive curative therapies and have improved overall survival compared with older patients. Studies should examine reasons for these differences and test interventions to mitigate any contributing failures in healthcare delivery processes.

## Abbreviations

BCLC, Barcelona Clinic Liver Cancer; HCC, hepatocellular carcinoma; HR, hazard ratio; OR, odds ratio; RR, risk ratio.

## Financial support

AGS’s research is conducted with support from the 10.13039/100000054National Cancer Institute (R01 CA212008, R01 CA222900, and R01 MD012565) and 10.13039/100004917Cancer Prevention Research Institute of Texas (RP200554). DD’s research is conducted with support from 10.13039/100004917CPRIT RP210041. The content is solely the responsibility of the authors and does not necessarily represent the official views of the National Institutes of Health, Cancer Prevention Research Institute of Texas, or the United States government.

## Authors’ contributions

Conceptualization: AGS. Data curation: OB, DD. Formal analysis: DD. Writing—original draft: OB, DD, AGS. Writing—review and editing: all authors. Have read and approved the final version of the manuscript for submission: all authors.

## Data availability statement

No new data were generated as part of this study. All new analyses are presented in the manuscript.

## Conflicts of interest

AGS has served as a consultant or on advisory boards for Genentech, AstraZeneca, Eisai, Exelixis, Bayer, Boston Scientific, Sirtex, Histosonics, FujiFilm Medical Sciences, Exact Sciences, Roche, Glycotest, Freenome, and GRAIL. NDP has served as a consultant or on advisory boards for Eisai, Exelixis, FujiFilm Medical Sciences, Sirtex, AstraZeneca, and Gilead. NER has served as consultant or on advisory boards for AstraZeneca, Eisai, Exelixis, and Genentech. AP is on the medical advisory board for Genentech, AstraZeneca, and Replimune. JDY provides a consulting service for AstraZeneca, Eisai, Exact Sciences, and FujiFilm Medical Sciences. None of the other authors have any relevant conflicts of interest to disclose.

Please refer to the accompanying ICMJE disclosure forms for further details.

## Supplementary data

[Multimedia component 1] Multimedia component 1

[Multimedia component 2] Multimedia component 2

[Multimedia component 3] Multimedia component 3

[Multimedia component 4] Multimedia component 4
