# Supplementary material for: Impact of age on clinical outcomes among patients with hepatocellular carcinoma: A systematic review and meta-analysis
Source: JHEP Rep. 2025 Feb 26;7(6):101368. doi: 10.1016/j.jhepr.2025.101368 (PMC12174979; doi:10.1016/j.jhepr.2025.101368)
Supplement: [Multimedia component 1] [file mmc1.pdf]

Impact of age on clinical outcomes among patients with hepatocellular carcinoma: A systematic review and meta-analysis

Olgert Bardhi, Darine Daher, Mausam Patel, Karim Seif El-Dahan, Nicole E. Rich, Sukul Mitta, Neehar D. Parikh, Anjana Pillai, Laura M. Kulik, Ju Dong Yang, Anand V. Kulkarni, Purva Gopal, Amit G. Singal

Table of contents

Fig. S1……………………………………………………………..……………………… 2

Fig. S2………………………………………………………………………………………….3

Fig. S3………………………………………………………………………………………….4

Fig. S4………………………………………………………………………………………….5

Fig. S5………………………………………………………………………………………….6

Table S1……………………………………………………………………………………....7

Table S2…………………….…………………………………………………………………..11

Table S3…………………….…………………………………………………………………..16

Table S4…………………….…………………………………………………………………..17

Table S5…………………….…………………………………………………………………..19

Table S6…………………….…………………………………………………………………..25

Table S7…………………….…………………………………………………………………..26

Fig. S1. Flow Diagram of Study Selection Process

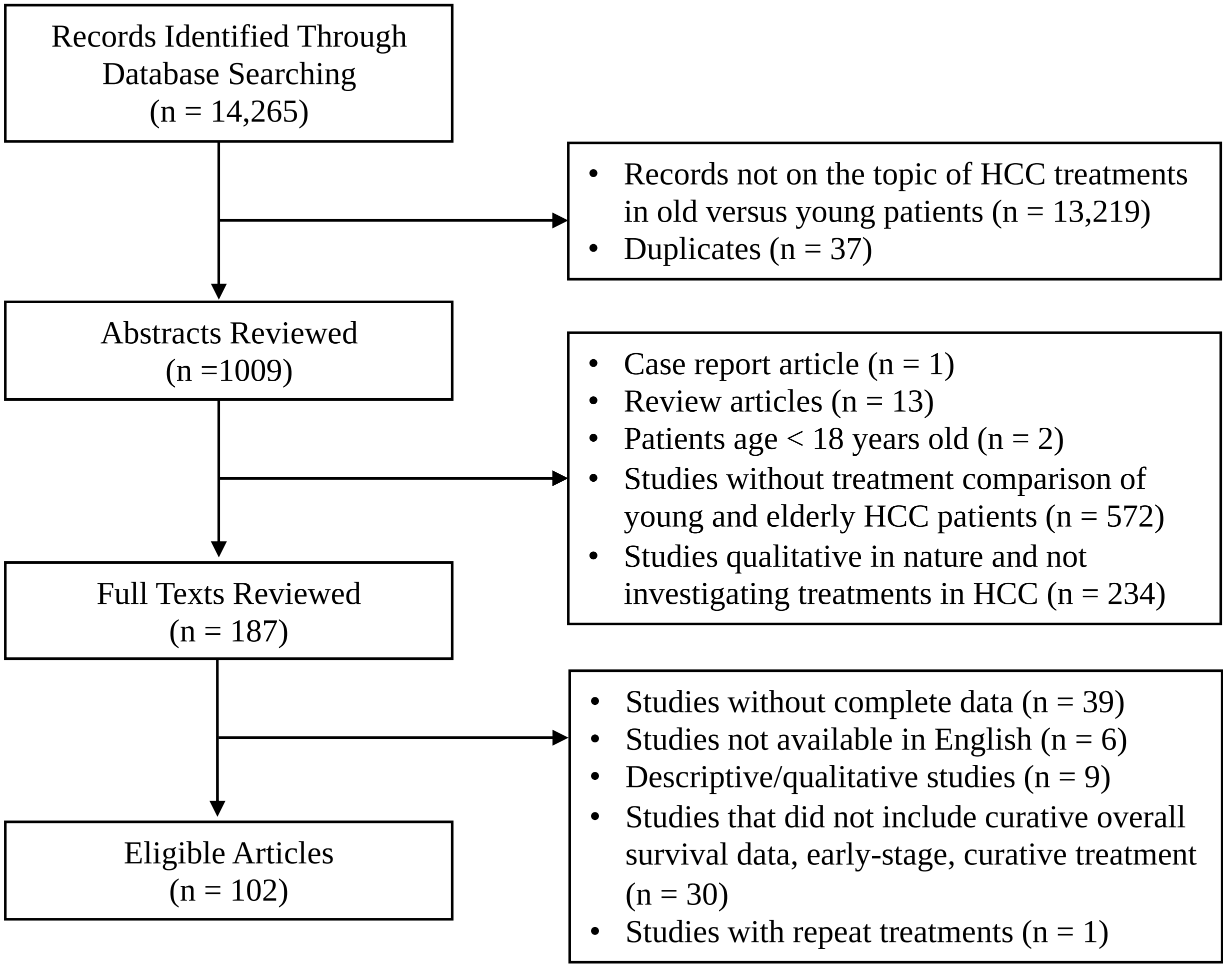

Fig. S2. Early-stage presentation between younger vs. older patients

Study	Experimental Events	Experimental Total	Control Events	Control Total	RR	95%-CI	Weight
Liu, Po-Hong, 2014	885	2270	300	812	1.06	[0.95; 1.17]	11.7%
Yan H et al, 2022	129	207	929	1395	0.94	[0.84; 1.05]	11.2%
Guo H et al, 2017	362	1212	86	318	1.10	[0.90; 1.35]	6.5%
Borzio et al, 2017	255	527	274	542	0.96	[0.85; 1.08]	10.6%
Kozyreva Olga et al, 2011	68	240	24	95	1.12	[0.75; 1.67]	2.3%
Lee Sang H, 2011	65	149	39	113	1.26	[0.92; 1.73]	3.4%
Tseng Po-Lin et al, 2010	35	55	16	33	1.31	[0.88; 1.97]	2.2%
Mirici-Cappa Federica et al, 2009	276	994	205	563	0.76	[0.66; 0.88]	8.9%
Chang Pik-Eu et al, 2008	1	55	3	583	3.53	[0.37; 33.40]	0.1%
Tsukioka G et al, 2006	129	234	48	91	1.05	[0.83; 1.31]	5.5%
Dohmen K et al, 2004	323	668	21	36	0.83	[0.62; 1.10]	3.9%
Lam C-M et al, 2004	17	121	209	1742	1.17	[0.74; 1.85]	1.8%
Giannini et al, 2002	6	24	14	51	0.91	[0.40; 2.08]	0.6%
Fan C et al, 2013	96	531	31	164	0.96	[0.66; 1.38]	2.7%
Kim Yun Jung et al, 2012	161	813	33	179	1.07	[0.77; 1.51]	3.0%
Ratana-Amornpin S et al, 2021	33	87	37	97	0.99	[0.69; 1.44]	2.6%
Rich N et al, 2019	345	849	117	261	0.91	[0.77; 1.06]	8.4%
Hori et al, 2014	169	282	76	140	1.10	[0.92; 1.32]	7.3%
Pignata Sandro et al, 2006	96	492	29	158	1.06	[0.73; 1.55]	2.5%
Ozenne et al, 2011	22	40	19	40	1.16	[0.75; 1.78]	2.0%
Seo et al, 2019	82	346	35	204	1.38	[0.97; 1.97]	2.8%
**Random effects model**		**10196**		**7617**	**1.00**	**[0.94; 1.07]**	**100.0%**

Heterogeneity: $I^2 = 35\%$, $\tau^2 = 0.0065$, $p = 0.06$

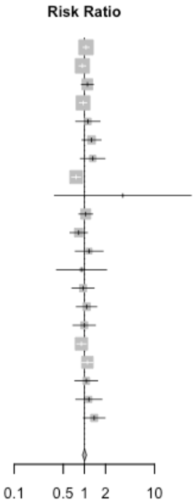

There was no significant difference in early-stage HCC presentation between young versus older patients (RR 1.00; 95%CI 0.94 – 1.07).

Fig. S3. Overall survival between younger vs. older patients

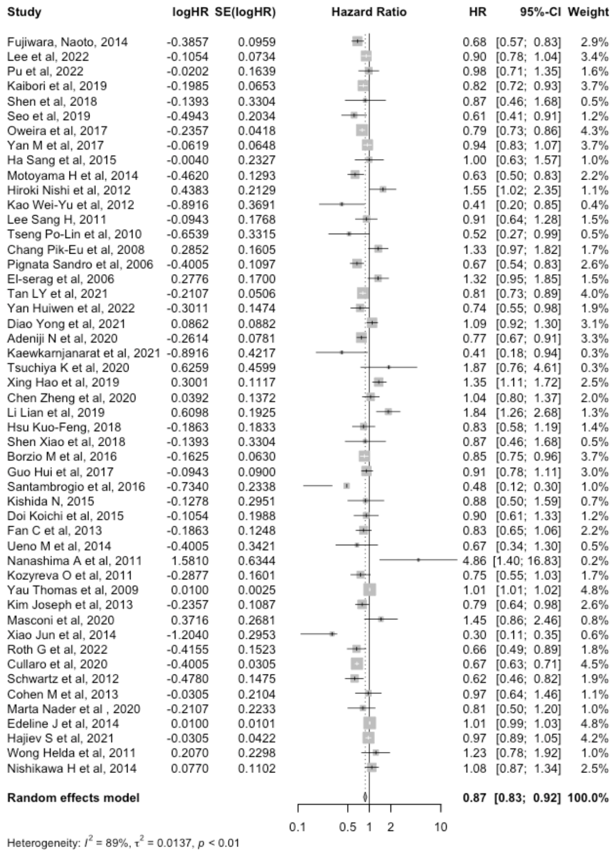

Younger patients had better overall survival than older patients (HR 0.87; 95%CI 0.83 – 0.92).

Fig. S4. Five-year survival between younger vs. older patients who underwent curative treatment.

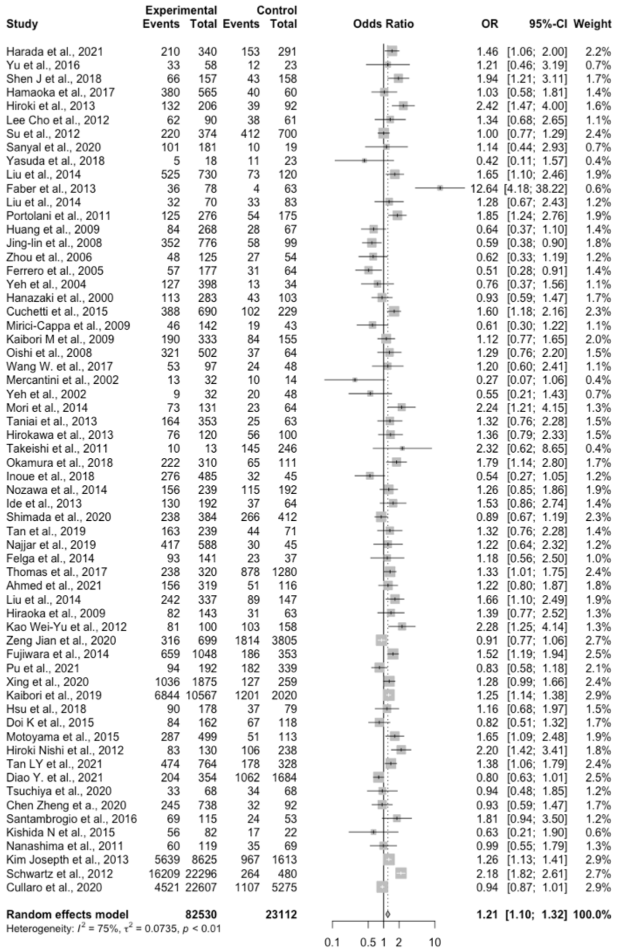

Younger patients who underwent curative treatment showed higher odds of 5-year survival compared to older patients (OR 1.21; 95%CI 1.10 – 1.32).

Fig. S5. Liver and Non-Liver Related Mortality in Younger and Older Patients

<u>Liver Related Mortality</u>

Study	Experimental Events	Experimental Total	Control Events	Control Total	RR	95%-CI	Weight
Lee Sang H et al, 2011	62	113	68	149	1.20	[0.94; 1.53]	32.9%
Tsukioka G et al, 2006	48	91	108	234	1.14	[0.90; 1.45]	33.3%
Kozyreva et al, 2011	46	95	140	240	0.83	[0.66; 1.05]	33.8%
**Random effects model**		**299**		**623**	**1.04**	**[0.83; 1.31]**	**100.0%**

Heterogeneity: $I^2 = 64\%$, $\tau^2 = 0.0263$, $p = 0.06$

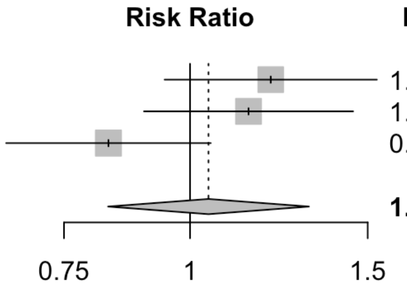

<u>Non-Liver Related Mortality</u>

Study	Experimental Events	Experimental Total	Control Events	Control Total	RR	95%-CI	Weight
Lee Sang H et al, 2011	2	113	4	149	0.66	[0.12; 3.54]	26.2%
Tsukioka G et al, 2006	48	91	9	234	13.71	[7.02; 26.79]	36.5%
Kozyreva et al, 2011	21	95	20	240	2.65	[1.51; 4.66]	37.3%
**Random effects model**		**299**		**623**	**3.36**	**[0.76; 14.74]**	**100.0%**

Heterogeneity: $I^2 = 90\%$, $\tau^2 = 1.4433$, $p < 0.01$

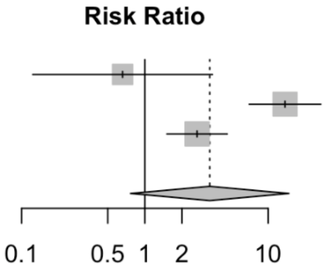

There was no difference in liver related mortality in older and younger patients (RR 1.04, 95%CI 0.83 – 1.31; I^2=64%). However, non–liver-related deaths were higher among older patients than younger patients (RR 3.36 ,95%CI 0.76–14.74; I^2=90%).

Table S1. Characteristics of all-comer studies investigating curative treatments.

Study, Year	Country	Cohort	Treatment	Age Cutoff	Number of Patients	Staging System
**Liu, Po-Hong, 2014**	Taiwan	Elderly: HCV 34%, HBV 36%<br><br>Young: HCV 29%, HBV 61%	Surgical Resection, RFA	< 75<br>≥ 75	3082	BCLC Stage 0 - A
**Yan H et al., 2022**	China	Elderly: HBV 88.2%, HCV 4.9%<br><br>Young: HBV 96.1%, HCV 0.5%	Surgical Resection	≤ 45<br>> 45	1602	BCLC Stage 0 - B
**Seo et al., 2019**	Korea	>Elderly: HBV 3.9%, HCV 37.3%<br><br>Elderly: HBV 15%, HCV 30.1%<br><br>Young: HBV 19.4%, HCV 25.7%	Surgical Resection, RFA	≥ 85<br>80 - 85<br>75 - 80 (cut off)	550	BCLC Stage 0 - A
**Oweira et al., 2017**	USA	N/A	Electrocautery, cryosurgery, laser, PEI, RFA, acetic acid injection.	70 - 80<br>> 80	6693	TNM Stage 1 & 2
**Borzio et al., 2017**	Italy	Elderly: HBV 9.2%, HCV 61.6%<br><br>Young: HBV 18.6%, HCV 49.3%	Surgical Resection, OLT, RFA	> 70<br>≤ 70	1069	BCLC Stage 0 - A
**Yan M et al., 2017**	USA	N/A	Surgical Resection, OLT, PEI, RFA	<50 (reference)<br>50-59<br>60 - 69	48347	Milan

				> 70 (cutoff)		
**Lee Sang H., 2011**	South Korea	Elderly: HBV 45.1%, HCV 23.9%<br><br>Young: HBV 75.8%, HCV 5.4%	Surgical Resection, OLT, RFA, PEI	< 65<br>≥ 65	262	BCLC Stage 0 - A
**Tseng Po-Lin et al., 2010**	Taiwan	Elderly: HCV 69.7%<br><br>Young: HCV 81.8%	Surgical Resection, RFA, PEI	< 70<br>≥ 70	88	BCLC Stage 0 - A
**Mirici-Cappa Federica et al., 2009**	Italy	Elderly: HBV 7.4%, HCV 62.8%<br><br>Young: HBV 11.9%, HCV 47.4%	Surgical Resection, RFA	< 70<br>≥ 70	1718	CLIP
**Chang Pik-Eu et al., 2008**	Singapore	Elderly: HCV 59.7%<br><br>Young: HCV 85.5%	Surgical Resection, RFA	≤ 40<br>> 40	638	TNM Stage 1
**Pignata Sandro et al., 2006**	Italy	Elderly: HBV 13%, HCV 84%<br><br>Young: HBV 23%, HCV 77%	Surgical Resection, PEI	< 70<br>≥ 70	650	CLIP
**Tsukioka G et al, 2006**	Japan	Elderly: HBV 3.2%, HCV 73.6%<br><br>Young: HBV 15.3%, HCV 77.7%	OLT, RFA	≥ 80<br>50 - 60	325	TNM Stage 1 & 2
**El-serag et al, 2006**	USA	N/A	Surgical Resection, RFA, OLT	>65 (cut off)<br>65-74<br>75+	2963	N/A

**Dohmen K et al., 2004**	Japan	Elderly: HBV 2.7%, HCV 83.3%<br><br>Young: HBV 10.1%, HCV 85.9%	Surgical Resection, PEI, RFA	< 80<br>≥ 80	704	N/A
**Lam C-M et al., 2004**	Hong Kong	Elderly: HBV 100%<br>Young: HBV 100%	Surgical Resection	≤ 40<br>> 40	1844	TNM Stage 1 & 2
**Giannini et al., 2002**	Italy	Elderly: HCV 100%<br><br>Young: HCV 100%	Surgical Resection, OLT, PEI, PEI+TACE	< 65<br>≥ 65	75	CLIP
**Guo Hui et al., 2017**	China	Elderly; HBV 49.4%, HCV 15.1%<br><br>Young: HBV 79.7%, HCV 3.2%	Surgical Resection, OLT, RFA, RFA/TACE/PEI	< 65<br>≥ 65	1530	BCLC Stage 0-A
**Fan C et al., 2013**	China	Elderly: HCV 23.8%, HBV 40.9%<br><br>Young: HCV 5.6%, HBV 76.6%	Surgical Resection, RFA	< 70<br>≥ 70	695	BCLC Stage 0 - A
**Kim Yun Jung et al., 2012**	South Korea	Elderly: HCV 26.3%, HBV 31.3%<br><br>Young: HCV 9.2%, HBV 69.2%	Surgical Resection, TACE, RFA/PEIT, Chemotherapy, Palliative	< 70<br>≥ 70	992	BCLC Stage 0 - A
**Kozyreva O et al., 2011**	USA	Elderly: HBV 14.7%, HCV 21.1%<br><br>Young: HBV 21.2%, HCV 48.3%	Surgical Resection, RFA, TACE, Radiation, OLT, Surgical Resection	< 70<br>≥ 70	335	TNM Stage 1

**Ratana-Amornpin S et al., 2021**	Thailand	Elderly: HBV 26.6%, HCV 25.5%<br><br>Young: HBV 54.9%, HCV 28.1%	Surgical Resection, RFA	< 65<br>≥ 65	184	BCLC Stage 0 - A
**Ozenne et al., 2011**	France	Elderly:<br>HBV 12.5%, HCV 57.5%<br><br>Young:<br>HBV 30%, HCV 32.5%	OLT, surgical resection, RFA	> 75<br>< 75	80	BCLC Stage A
**Hori et al., 2014**	Japan	Young:<br>HBV 11.3%, HCV 80.8%<br><br>Elderly:<br>HBV 3.5%, HCV 80.7%	Surgical Resection, RFA, RFA/TACE	< 75<br>≥ 75	422	TNM Stage 1 & 2

Abbreviations: HBV, hepatitis B virus; HCV, hepatitis C virus; RFA, radiofrequency ablation; TACE, transarterial chemoembolization; OLT, orthotopic liver transplantation; PEI, percutaneous ethanol injection; BCLC, Barcelona Clinic Liver Cancer; TNM, tumor node metastasis; CLIP, cancer of the liver Italian program.

Table S2. Studies reporting overall survival among patients who underwent curative treatment

Study, Year	Country	Age Cut-off	Number of Patients	Curative Treatment	Overall Survival		Significance (younger vs. older)
					Younger 1-, 3-, 5-year	Older 1-, 3-, 5-year	
**Harada et al., 2021**	Japan	< 70<br>≥ 70	< 70, n= 340<br>≥ 70, n= 291	Resection	92.8%, 76%, 61.8%	89.1%, 72.9%, 52.9%	**p< 0.01**
**Yu et al., 2016**	China	< 70<br>≥ 70	≤ 70, n= 58<br>> 70, n= 23	Resection	79.1%, 61.1%, 57.1%	82.6%, 73.9%, 56.5%	p= 0.75
**Shen J et al., 2018**	China	≤ 55<br>> 55	≤ 55, n= 157<br>> 55, n= 158	Resection	86.5%, 57.5%, 42.4%	78.1%, 45.1%, 27.4%	**p= 0.007**
**Hamaoka et al., 2017**	Japan	≥80<br><80	≥ 80, n= 60<br>< 80, n=565	Resection	99.5%, 80.3%, 67.4%	92.7%, 77.5%, 67.1%	p= 0.62
**Hiroki et al., 2013**	Japan	≥ 75<br>< 75	≥ 75, n= 92<br>< 75, n= 206	Resection	91.0%, 77.5%, 64.4%	90.0%, 73.3%, 43.0%	p= 0.19
**Lee Cho et al., 2012**	South Korea	< 70<br>≥ 70	≤ 40, n= 90<br>≥ 70, n= 61	Resection	94.4%, 75.1%, 69.1 %	86.5%, 75.1%, 62.3%	NS*
**Su et al., 2012**	Taiwan	≤ 55<br>> 55	≤ 55, n= 374<br>> 55, n= 700	Resection	82.4%, 67.3%, 58.9%	86.7%, 66.4%, 51.4%	**p= 0.02****
**Sanyal et al., 2020**	UK	≥80<br><80	≥ 80, n= 19<br>< 80, n= 181	Resection	83.4%, 65.7%, 56.3%	88.2%, 81.9%, 55.8%	p= 0.83
**Yasuda et al., 2018**	Japan	< 70<br>≥ 70	< 70, n = 18<br>≥ 70, n= 23	Resection	83.3%, 42.8%, 30.6%	82.6%, 50.6%, 50.6%	p= 0.40
**Liu et al., 2014**	Taiwan	≥ 75<br>< 75	≥ 75, n= 129<br>< 75, n= 730	Resection	92%, 82%, 72%	93%, 82%, 61%	**p= 0.02****
**Faber et al., 2013**	German y	< 70<br>≥ 70	< 70, n= 78<br>≥ 70, n= 63	Resection	78.5%, 56.5%, 47.1%	59.9%, 40.3%, 6.7%	**p< 0.001**
**Liu et al., 2014**	China	< 40<br>> 40	< 40, n= 70<br>> 40, n= 83	Resection	93%, 79%, 47%	85%, 75%, 40%	p= 0.11
**Portolani et al., 2011**	Italy	< 70<br>≥ 70	< 70, n= 276<br>≥ 70, n= 175	Resection	94.1%, 68.6%, 45.4%	83.7%, 62.7%, 31.4%	NS*
**Huang et al., 2009**	China	< 70<br>≥ 70	< 70, n= 268<br>≥ 70, n= 67	Resection	71.6%, 39.9%, 31.4%	83.3%, 54.6%, 43.2%	p= 0.16
**Jing-lin et al., 2008**	China	< 65<br>≥ 65	< 65, n= 776<br>≥ 65, n= 99	Resection	77.8%, 53.4%, 45.4%	84.6%, 65.3%, 59.5%	p= 0.12
**Zhou et al., 2006**	China	< 65<br>≥ 65	< 65, n= 125<br>≥ 65, n= 54	Resection	79.6%, 49.1%, 38.6%	88.8%, 56.8%, 50.1%	p= 0.38

**Ferrero et al., 2005**	Italy	< 70<br>≥ 70	≤ 70, n= 177<br>> 70, n= 64	Resection	74.1%, 49. 6%, 32.3%	81.3%, 57.1%, 48.6%	p= 0.08
**Yeh et al., 2004**	Taiwan	< 70<br>≥ 70	< 70, n= 398<br>≥ 70, n= 34	Resection	69%, 45.5%, 32.1%	85.3%, 64.3%, 39.6%	p= 0.32
**Hanazaki et al., 2000**	Japan	< 70<br>≥ 70	< 70, n= 283<br>≥ 70, n= 103	Resection	85.1%, 55.2%, 40.0%	83.4%, 51.0%, 42.2%	p= 0.66
**Cuchetti et al., 2015**	Italy	< 60<br>60 - 66<br>67 - 70<br>> 70	< 60, n= 229<br>60 – 66, n= 230<br>67- 70, n=231<br>> 70, n= 229	Resection	<60: 86%, 67%, 57%<br>60 - 66: 92%, 68%, 56%<br>67 - 70: 90%, 69%, 54%	>70: 89%, 68%, 44%	p= 0.40
**Mirici-Cappa et al., 2009**	Italy	< 70<br>≥ 70	< 70, n= 142<br>≥ 70, n= 43	Resection	86.5%, 61.6%, 32.4%	95.2%, 67.3%, 44.8%	p= 0.08
**Kaibori M et al., 2009**	Japan	< 70<br>≥ 70	< 70, n= 333<br>≥ 70, n= 155	Resection	*3-, 5-, 7-year OS:*<br>69.7%, 57.3%, 44.0%	*3-, 5-, 7-year OS:*<br>70.3%, 54.6%, 35.8%	p= 0.79
**Oishi et al., 2008**	Japan	< 75<br>≥ 75	< 75, n= 502<br>≥ 75, n= 64	Resection	*3-, 5-, 10-year OS:*<br>81%, 64%, 33%	*3-, 5-, 10-year OS:*<br>77%, 58%, 32%	NS*
**Wang W. et al., 2017**	China	< 70<br>≥ 70	< 70, n= 97<br>≥ 70, n= 48	Resection	*5-year OS*<br>55%	*5-year OS*<br>52%	p= 0.61
**Mercantini et al., 2002**	Italy	< 65<br>≥ 65	< 65, n= 32<br>≥ 65, n= 14	Resection	*5-year OS:*<br>41.8%	*5-year OS:*<br>77.4%	p= 0.30
**Yeh et al., 2002**	Taiwan	≤ 30<br>> 70	≤ 30, n= 32<br>> 70, n= 48	Resection	*1-, 5-year OS:*<br>52.2%, 30.4%	*1-, 5-year OS:*<br>84.4%, 42.1%	p= 0.08
**Mori et al., 2014**	Japan	< 70<br>≥ 70	< 70, n= 131<br>≥ 70, n= 64	Resection	*5-year OS:*<br>56.3%	*5-year OS:*<br>36.0%	**p= 0.01**
**Taniai et al., 2013**	Japan	≥ 75<br>< 75	≥ 75, n= 63<br>< 75, n= 353	Resection	*3-, 5- year OS:*<br>63.4%, 46.6%	*3-, 5- year OS:*<br>56.2%, 40.2%	p= 0.62
**Hirokawa et al., 2013**	Japan	< 70<br>≥ 70	< 70, n= 120<br>≥ 70, n= 100	Resection	93%, 79%, 64%	91%, 71%, 56%	p= 0.07
**Takeishi et al., 2011**	Japan	< 40<br>≥ 40	< 40, n= 13<br>≥ 40, n= 246	Resection	*5-, 10-year OS:*<br>84.6%, 55.9%	5-, 10-year OS:<br>59.2%, 30.7%	p= 0.06
**Okamura et al., 2018**	Japan	< 75<br>≥ 75	< 75, n= 310<br>≥ 75, n= 111	Resection	*3-year, 5-year OS:*<br>77.4%, 71.9%	3-year, 5-year OS:<br>77.7%, 59.0%	p= 0.31
**Inoue et al., 2018**	Japan	≥80<br><80	≥ 80, n= 45<br>< 80, n= 485	Resection	85.3%, 70.9%, 57.0%	87.8%, 81.0%, 72.9%	p= 0.48

**Tsujita 2 et al., 2012**	Japan	>80<br><80	> 80, n= 23<br>< 80, n= 385	Resection	*3-year OS:*<br>84.8%	*3-year OS:*<br>95.7%	p= 0.56
**Nozawa et al., 2014**	Japan	< 70<br>70 – 80<br>> 80	< 70, n= 239<br>70 – 80, n = 172<br>> 80, n= 20	Resection	*5-year OS*<br>65.3%	*5-year OS*<br>70 - 80: 59.5%<br>> 80: 66.9%	p=0.44
**Ide et al., 2013**	Japan	> 75<br>< 75	> 75, n=64<br>< 75, n= 192	Resection	*5-year OS*<br>68%	*5-year OS*<br>58%	p=0.64
**Shimada et al., 2020**	Japan	> 80<br>65 - 79<br>< 65	> 80, n= 49<br>65 – 79, n= 363<br>< 65, n= 384	Resection	*5-year OS:*<br>< 65: 62%	*5-year OS:*<br>> 80: 62%<br>65- 79: 65%	p=0.86
**Tan et al., 2019**	Australi a	< 70<br>> 70	< 70, n= 239<br>> 70, n= 71	Resection	*5-year OS:*<br>68.5%	*5-year OS:*<br>62%	p=0.71
**Najjar et al., 2019**	USA	< 70<br>> 70	< 70, n= 588<br>> 70, n= 45	Liver transplant	*5-year OS:*<br>71%	*5-year OS:*<br>67%	p=0.35
**Felga et al., 2014**	Brazil	< 65<br>> 65	< 65, n= 141<br>> 65, n= 37	Liver transplant	81%, 70%, 66%	78%, 64%, 64%	p= 0.49
**Thomas et al., 2017**	USA	< 40<br>> 40	< 40, n= 320<br>> 41, n= 1,280	Liver transplant	*5-year OS*<br>74.4%	*5-year OS*<br>68.6%	p= 0.09
**Ahmed et al., 2021**	USA	< 65<br>> 65	< 65, n= 319<br>> 65, n= 116	Liver transplant	86.0%, 64.8%, 49.0%	82.5%, 59.0%, 44.8%	p= 0.13
**Liu et al., 2014**	Taiwan	> 75<br>< 75	> 75, n= 147<br>< 75, n= 337	Local ablation	95%, 81%, 72%	96%, 78%, 61%	p= 0.69
**Hiraoka et al., 2009**	Japan	< 75<br>> 75	< 75, n= 143<br>> 75, n= 63	Local ablation	93.2%, 78.3%, 57.5%	91.7%, 82.5%, 49.7%	p= 0.60
**Kao Wei-Yu et al., 2012**	Taiwan	< 65<br>> 65	< 65, n= 100<br>> 65, n= 158	Local ablation	98.0%, 87.0%,81.3%	93.9%, 72.4%, 65.4%	p= 0.01
**Zeng Jian et al., 2020**	China	< 40<br>> 40	< 40, n= 699<br>> 40, n= 3,805	Resection	81.2%, 61%, 45.3%	86.4%,65.3%, 47.7%	p= 0.01
**Fujiwara et al., 2014**	Japan	> 75<br>< 75	> 75, n= 353<br>< 75, n= 1,048	Local ablation	*Mortality Rate*<br>2.7%, 17.7%, 37.1%	*Mortality Rate*<br>4.5%, 24.4%, 47.3%	p< 0.001
**Pu et al., 2021**	China	< 35<br>> 70	< 35, n= 192<br>> 70, n= 339	Resection	80.2%, 62.2%, 49.1%	89%, 71.1%, 53.9%	p> 0.05
**Xing et al., 2020**	China	> 70<br>< 70	> 70, n= 259<br>< 70, n= 1,875	Resection	86.7%, 67.5%, 55.3%	89.2%, 69.6%, 49.4%	p= 0.03
**Kaibori et al., 2019**	Japan	40 - 59<br>60 - 74	40 - 59, n= 2,991	Resection	*5-year OS*<br>40 - 59: 68.8%	*5-year OS*<br>>75: 59.5%	p< 0.001

		≥ 75	60 - 74, n= 7,576<br>≥ 75, n= 2,020		60 - 74: 63.2%		
**Hsu et al., 2018**	Japan	< 70<br>≥ 70	< 70, n= 178<br>≥ 70, n= 79	Resection	79%, 57%, 51%	76%, 55%, 48%	p= 0.32
**Doi K et al., 2015**	Japan	< 70<br>≥ 70	< 70, n= 162<br>≥ 70, n= 118	Local ablation	*3-, 5-year OS*<br>70%, 52%	*3-, 5-year OS*<br>73%, 57%	p= 0.90
**Motoyama et al., 2015**	Japan	< 75<br>≥ 75	< 75, n= 499<br>≥ 75, n= 113	Resection	*5-year OS*<br>57.6%	*5-year OS*<br>46%	p= 0.02
**Hiroki Nishi et al., 2012**	Japan	< 75<br>≥ 75	< 75, n= 238<br>≥ 75, n= 130	Local ablation	97.6%, 83.7%, 64.0%	90.0%, 64.1%, 44.8%	p= 0.001
**Tan LY et al., 2021**	Singapore	< 70<br>70 - 79<br>≥ 80	< 70, n= 764<br>70 – 79, n= 278<br>≥ 80, n= 50	Resection	*1-, 5-, 10-years OS*<br><70: 91.5%, 62.1%, 46.0%	*1-, 5-, 10-years OS*<br>>80: 77.9%, 37.7%, 10.5%<br>70-79: 85.9%, 57.7%, 27.4%	<70 vs 70-79: p= 0.03<br><br>70-79 vs >80: p= 0.05
**Diao Y. et al., 2021**	China	< 40<br>≥ 40	< 40, n= 354<br>≥ 40, n= 1,684	Resection	83.2%, 67.2%, 57.7%	91.9%, 75%, 63.1%	p= 0.31
**Tsuchiya et al., 2020**	Japan	< 80<br>≥ 80	< 80, n= 68<br>≥ 80, n= 68	Local ablation	94.1%, 72.8%, 49.3%	98.5%, 87.9%, 50.5%	p= 0.83
**Chen Zheng et a., 2020**	China	< 65<br>≥ 65	< 65, n= 738<br>≥ 65, n= 92	Resection	68.9%, 42.0%, 33.2%	71.3%, 43.2%, 35.0%	p= 0.74
**Santambrogio et al., 2016**	Italy	< 75<br>≥ 75	< 75, n= 115<br>≥ 75, n= 53	Resection	*3-, 5-year OS*<br>82%, 60%	*3-, 5-year OS*<br>65%, 46%	p= 0.02
**Kishida N et al., 2015**	Japan	< 75<br>≥ 75	< 75, n= 82<br>≥ 75, n= 22	Resection	*5-year OS*<br>69%	*5-year OS*<br>81%	p= 0.35
**Nanashima et al., 2011**	Japan	< 50<br>50 - 69<br>70 - 79<br>≥ 80	< 50, n= 9<br>50 – 69, n= 110<br>70 – 79, n= 57<br>≥ 80, n= 12	Resection	*5-, 10-year OS*<br>< 50: 50%, 25%<br>50 - 69: 51%, 27%	*5-, 10-year OS*<br>70 - 79: 46%, 36%<br>> 80: 78%, N/A	p= 0.82
**Kim Josepth et al., 2013**	USA	35 - 49<br>50 - 64<br>≥ 65	35 - 49<br>50 - 64<br>≥ 65	Liver transplant	*1-, 5-year OS*<br>35 - 49: 89%, 67%<br>50 - 64: 87%, 65%	*1-, 5-year OS*<br>> 65: 85%, 60%	p= 0.001
**Schwartz et al., 2012**	USA	< 70<br>≥ 70	< 70, n= 22,296<br>≥ 70, n= 480	Liver transplant	*5-year OS*<br>72.7%	*5-year OS*<br>55.2%	N/A

**Cullaro et al., 2020**	USA	< 65<br>$\geq$ 65	< 65, n= 22,607<br>$\geq$ 65, n= 5,275	Liver transplant	5-year post-transplant mortality<br>20%	5-year post-transplant mortality<br>21%	p= 0.02

Abbreviations: OS, overall survival; NS, not significant.
*P-value not explicitly stated
**Not significant after study propensity score matching.

Table S3. Clinical Outcomes in Younger and Older Patients, Stratified by Study Period

Category	Younger Patients (%)	Older Patients (%)
**Curative Treatment Receipt**		
All studies	50.4 (40.0-60.6)	38.0 (27.6-48.4)
<2005	47.8 (9.9-85.7)	46.7 (4.6-88.8)
2005-2009	49.4 (14.1-84.6)	22.3 (6.0-38.6)
2010-2014	58.8 (39.7-77.9)	45.9 (22.8-69)
>2015	42.5 (18.4-66.6)	36.4 (12.3-60.6)
**Early-Stage HCC**		
All studies	36.3 (28.8-43.7)	35.0 (27.6-42.4)
<2005	29.1 (9.3-49.0)	32.6 (5.9- 59.2)
2005-2009	26.1 (4.3-47.8)	27.0 (4.9-49.1)
2010-2014	40.9 (28.6-53.2)	35.5 (26.0-45.1)
>2015	40.5 (29.5-51.4)	40.7 (26.7-54.7)

Table S4. Studies reporting differences in performance status, liver function, comorbidities, and curative treatment receipt in younger versus older patients.

Study, Year	Country	Age Cutoff	Performance Status*	Liver Function**	Comorbidities[γ]	Curative Treatment Receipt
**Liu et al., 2014**	Taiwan	< 75<br>≥ 75	Older < Younger	No difference	N/A	Older < Younger
**Yan H. et al., 2022**	China	≤ 45<br>> 45	N/A	Older < Younger	N/A	Older < Younger
**Seo et al., 2019**	South Korea	75-80<br>80-85<br>≥ 85	No difference	No difference	No difference	Older < Younger
**Oweira et al., 2017**	USA	< 70<br>≥ 70	N/A	N/A	N/A	Older < Younger
**Borzio et al., 2016**	Italy	≤ 70<br>> 70	Older < Younger	Older < Younger	Older > Younger	Older < Younger
**Yan M. et al., 2017**	USA	Varied	N/A	N/A	N/A	Older < Younger
**Lee Sang H et al., 2011**	South Korea	< 65<br>≥ 65	Older < Younger	No difference	Older > Younger	Older < Younger
**Tseng Po-Lin et al., 2010**	China	< 70<br>≥ 70	N/A	N/A	N/A	Older < Younger
**Mirici-Cappa et al., 2009**	Italy	< 70<br>≥ 70	N/A	Older > Younger	Older > Younger	Older < Younger
**Chang et al., 2008**	Singapore	≤ 40<br>> 40	N/A	N/A	N/A	Older < Younger
**Pignata et al., 2006**	Italy	< 70<br>≥ 70	N/A	No difference	N/A	Older < Younger
**Tsukioka et al., 2006**	Japan	< 80<br>≥ 80	Older < Younger	No difference	N/A	Older < Younger
**El-Serag et al., 2006**	USA	Varied	N/A	N/A	No difference	Older < Younger
**Dohmen et al., 2004**	Japan	< 80<br>≥ 80	N/A	No difference	N/A	No difference
**Lam et al., 2004**	China	≤ 40<br>> 40	N/A	Older < Younger	Older > Younger	No difference

**Giannini et al., 2002**	Italy	< 65<br>≥ 65	N/A	No difference	N/A	No difference
**Guo et al., 2017**	China	< 65<br>≥ 65	Older < Younger	Older < Younger	Older > Younger	Older < Younger
**Fan et al., 2013**	China	< 70<br>≥ 70	Older < Younger	No difference	Older > Younger	Older < Younger
**Kim et al., 2012**	South Korea	< 70<br>≥ 70	No difference	No difference	Older > Younger	Older < Younger
**Kozyreva et al., 2011**	USA	< 70<br>≥ 70	Older < Younger	No difference	Older > Younger	Older < Younger
**Ratana-Amornpin et al., 2021**	Thailand	< 65<br>≥ 65	N/A	No difference	Older > Younger	No difference
**Ozenne et al., 2011**	France	< 75<br>≥ 75	No difference	No difference	N/A	Older < Younger
**Hori et al., 2014**	Japan	< 75<br>≥ 75	N/A	N/A	N/A	Older < Younger

Abbreviations: N/A, not applicable.
* Performance status using Eastern Cooperative Oncology Group (ECOG) when available.
** Liver function using Child-Pugh score when available.
[γ] Reported comorbidities may vary depending on study and available data, though typically included diabetes and hypertension.

Table S5. Studies reporting absolute overall survival estimates for younger and older patients

Author, Year	Type of treatment receipt	Number of Younger Patients	Number of Older Patients	Age cut-off used	Survival
**Fujiwara N. et al, 2014**	Local ablation	1,048	353	75 years	**1-, 3-, 5-year Mortality:**<br>Older: 4.5%, 24.4%, 47.3%<br>Young: 2.7%, 17.7%, 37.1%
**Lee et al, 2022**	Not specified	4,294	2,147	65 years	**1-, 3-, 5-year OS:**<br>Older: 59%, 45.5%, 33.5%<br>Young: 60%, 46.3%, 35.7%
**Pu et al, 2022**	Surgical resection	192	339	>70 vs. <35 years	**1-, 3-, 5-year OS:**<br>Older: 89%, 71.1%, 53.9%<br>Young: 80.2%, 62.2%, 49.1%
**Kaibori et al, 2019**	Surgical resection	10,567	2,020	75 years	**5-year OS:**<br>40 - 59 yr: 68.8%<br>60 - 74 yr: 63.2%<br>>75: 59.5%
**Seo et al., 2019**	Surgical resection	346	204	80 years	**Median OS:**<br>Older: 38.5 +/- 8.3 months<br>Young: 45.8 +/- 2.6 months
**Motoyama et a., 2014**	Surgical resection	499	113	75 years	**5-year OS:**<br>Older: 57.6%

					Young: 46%
**Hiroki Nishi et al., 2012**	Local ablation	238	130	75 years	**The 1-, 3- and 5-year OS**<br>Older: 90.0%, 64.1% and 44.8%<br>Young: 97.6%, 83.7% and 64.0%
**Kao Wei-Yu et al., 2012**	Local ablation	100	158	65 years	**1, 3, and 5-year OS**<br>Older: 93.9%, 72.4%, and 65.4%<br>Younger: 98.0%, 87.0%, and 81.3%
**Lee Sang et al., 2011**	Combined treatments	149	113	65 years	**1- and 3-year OS**<br>Older: 79.5% and 30.4%<br>Young: 68.7% and 39.5%
**Tseng et al., 2010**	Combined treatments	55	33	70 years	**4-year OS**<br>Young: 57.4%<br>Older: 28.9%
**Chang et al., 2008**	Combined treatments	55	583	40 years	**Median OS**<br>Young: 51.2 months<br>Older: 11.6 months
**Pignata et al., 2006**	Combined treatments	158	492	70 years	**1-year OS**<br>Older: 53.5%<br>Young: 60.2%
**Tan et al., 2021**	Surgical resection	764	328	70 years	**1-, 5-, 10-years OS**<br>>80: 77.9%, 37.7%, 10.5%

					70-79: 85.9%, 57.7%, 27.4%<br><70: 91.5%, 62.1%, 46.0%
**Diao et al., 2021**	Surgical resection	354	1,684	40 years	**1-, 3-, 5-year OS:**<br>Older: 91.9%, 75%, 63.1%<br>Young: 83.2%, 67.2%, 57.7%
**Tsuchiya K et al., 2020**	Local ablation	68	68	80 years	**1-, 3-, 5-year OS:**<br>Older: 98.5%, 87.9%, and 50.5%<br>Young: 94.1%, 72.8%, and 49.3%
**Xing Hao et al., 2019**	Surgical resection	1,875	259	70 years	**1-, 3-, 5-year OS:**<br>Older: 89.2%, 69.6%, 49.4%<br>Young: 86.7%, 67.5%, 55.3%
**Chen Zheng et al., 2020**	Surgical resection	738	92	65 years	**1-, 3-, 5-year OS:**<br>Older: 71.3%, 43.2%, 35.0%<br>Young: 68.9%, 42.0%, 33.2%
**Li Lian et al., 2019**	Surgical resection	84	84	60 years	**1-year and 5-year OS:**<br>Older: 73.9% and 37.3%<br>Young: 54.2% and 22.2%
**Hsu et al., 2018**	Surgical resection	178	79	70 years	**1-, 3-, 5-year OS:**<br>Older: 76%, 55%, and 48%<br>Young: 79%, 57%, and 51%
**Borzio et al., 2016**	Combined treatments	527	542	70 years	**Median OS:**

					Older: 29 months<br>Young: 33 months
**Guo Hui et al., 2017**	Combined treatments	1,212	318	65 years	**Median OS**<br>Older: 27 months<br>Young: 33 months
**Santambrogio et al., 2016**	Surgical resection	115	53	75 years	**3-, 5-year OS:**<br>Older: 65%, 46%<br>Young: 82%, 60%
**Kishida N et al., 2016**	Surgical resection	82	22	75 years	**5-year OS:**<br>Older: 81%<br>Young: 69%
**Doi K et al., 2015**	Local ablation	162	118	70 years	**3- and 5-year OS:**<br>Older: 73% and 57%<br>Young: 70% and 52%
**Fan et al., 2014**	Combined treatment	531	164	70 years	**Median OS:**<br>Older: 26.2 months<br>Young: 28.3 months
**Nanashima et al., 2011**	Surgical resection	119	69	70 years	**5-, 10-year OS:**<br>< 50: 50%, 25%<br>50 - 69: 51%, 27%<br>70 - 79: 46%, 36%<br>> 80: 78%

**Yau Thomas et al., 2009**	TACE	2,452	550	70 years	**1-, 3-, 5- year OS:**<br>Older: 54.4%, 23.2%, 10.6%<br>Young: 39.2% 14.9% 8.4%
**Kim Joseph et al., 2013**	Liver transplant	8,625	1,613	65 years	**1-, 5-year OS:**<br>35 - 49: 89%, 67%<br>50 - 64: 87%, 65%<br>> 65: 85%, 60%
**Xiao Jun et al., 2014**	TACE	1,877	616	60 years	**1- and 3-year OS**<br>Young: 31.8% and 6.5%<br>Older: 84.7% and 28.9%
**Roth G et al, 2022**	TACE	183	88	70 years	**Median OS**:<br>Older: 31.0 months<br>Young: 45.1 months
**Cullaro et al., 2020**	Liver transplant	22,607	5,275	65 years	**1-, 5- year post-OLT Mortality:**<br>Elderly: 10%, 21%<br>Young: 8%, 20%
**Schwartz et al., 2012**	Liver transplant	22,296	480	70 years	**1, 3, 5- year OS:**<br>Older: 83.0%, 67.3%, and 54.5%<br>Young: 89.4%, 76.7%, 67.8%
**Cohen M et al., 2013**	TACE	79	23	75 years	**1, 2, 3-year OS**<br><65: 74%, 37%, 31%<br>65-75: 83%, 66%, 48%

					≥75: 86%, 41%, 23%
**Hajiev et al., 2021**	Sorafenib	4,806	792	75 years	**Median OS**<br>Older: 7.3 (95% CI: 7.0–7.6) months<br>Young: 7.2 (95% CI: 6.4–8.0) months
**Wong Hilda et al., 2011**	Sorafenib	137	35	70 years	**Median OS (months)**<br>Older: 5.32 (4.44–10.35) months<br>Young: 5.16 (4.21–6.05) months
**Nishikawa H et al., 2014**	Sorafenib	279	179	75 years	**Median OS**<br>Older: 9.7 (95% CI, 7.5-12.0) months<br>Young: 8.2 (95% CI, 6.9-9.6) months

Abbreviations: OS, overall survival; TACE, transarterial chemoembolization

Table S6. Studies reporting liver vs. non-liver mortality for younger and older patients

Author, Year	Type of treatment receipt	Number of younger patient liver related deaths	Number of older patient liver-related deaths	Number of younger patient non-liver related deaths	Number of older patient non-liver related deaths
Lee et al., 2011	Any treatment	68/149	62/113	4/149	2/113
Kozyreva et al., 2011	Any treatment	140/240	46/95	20/240	21/95
Adeniji et al., 2020	Liver transplant	12/4001	11/979	20/4001	18/979
Kim et al., 2013	Liver transplant	875/8625	149/1613	1536/8625	377/1613
Pu et al, 2022	Surgical resection	11/192	93/339	100/192	96/339
Motoyama et a., 2014	Surgical resection	224/499	41/113	26/499	15/113
Santambrogio et al., 2016	Surgical resection	39/115	19/53	13/115	11/53
Fujiwara. et al, 2014	Local ablation			81/1048	52/353
Hiroki Nishi et al., 2012	Local ablation	51/238	27/130	7/238	13/130
Kao et al., 2012	Local ablation	8/100	31/158	2/100	4/158
Tsuchiya et al., 2020	Local ablation	16/68	17/68	11/68	7/68
Masconi et al., 2020	Chemoembolization	66/145	43/80	15/145	16/80
Yau et al., 2009	Chemoembolization	691/2452	145/550	29/2452	29/550

Table S7. Studies comparing outcomes between younger and older patients with early-stage HCC

Author, Year	Staging System	Type of treatment receipt	Age cut-off	Proportion of younger patients undergoing curative treatment	Proportion of older patients undergoing curative treatment
**Oweira et al, 2017**	TNM I & II	Surgical Resection, liver transplantation, local ablation	70 – 80 vs. > 80 years	1,876/4,563	581/2,130
**Yan H et al, 2022**	BCLC 0-B	Surgical Resection	45 years	12/207	43/1395
**Tseng et al, 2010**	BCLC 0-A	Surgical Resection, local alation	70 years	19/55	7/33*
**Yan M et al, 2017**	Milan	Surgical Resection, liver transplantation	50 years	Resection: 565/4,697<br>Transplant: 392/4,697	Resection: 3,740/37,791<br>Transplant: 2,119/37,791
**Guo H et al, 2017**	BCLC 0-A	Local ablation	65 years	86/1,212	32/318
**Tsukioka G et al, 2006**	TNM I & II	Liver transplantation, local ablation	50 – 60 vs. >80 years	N/R	N/R
**Lee et al, 2011**	BCLC 0-A	Surgical Resection, liver transplantation, local ablation	65 years	N/R	N/R
**Borzio et al, 2016**	BCLC 0-A	Surgical Resection, liver transplantation, local ablation	70 years	N/R	N/R
**Kim Yun et al, 2012**	BCLC 0-A	Surgical Resection, liver transplantation, local ablation	70 years	N/R	N/A
