# Supplementary material for: Impact of age on clinical outcomes among patients with hepatocellular carcinoma: A systematic review and meta-analysis
Source: JHEP Rep. 2025 Feb 26;7(6):101368. doi: 10.1016/j.jhepr.2025.101368 (PMC12174979; doi:10.1016/j.jhepr.2025.101368)
Supplement: [Multimedia component 4] [file mmc4.pdf]

JHEP|Reports

Impact of age on clinical outcomes among patients with hepatocellular carcinoma: A systematic review and meta-analysis

Olgert Bardhi[1,†], **Darine Daher**[1,†], Mausam Patel[1], Karim Seif El-Dahan[1], Nicole E. Rich[1], Sukul Mitta[1], Neehar D. Parikh[2], Anjana Pillai[3], Laura M. Kulik[4], Ju Dong Yang[5], Anand V. Kulkarni[6], Purva Gopal[1], Amit G. Singal[1,*]

JHEP Reports **2025**. vol. 7 | 1–9

Check for updates

Background & Aims: Older adults have lower treatment eligibility and worse survival across cancer types; however, the association between age and outcomes in patients with hepatocellular carcinoma (HCC) has not been well characterized.

Methods: We performed a search of the PubMed, Ovid MEDLINE, and EMBASE databases from January 2000 to July 2022 to identify studies reporting tumor stage, curative treatment, and overall survival among patients with HCC, stratified by age. Using the DerSimonian and Laird method for a random-effects model, we calculated pooled risk ratios (RRs) for curative treatment receipt and hazard ratios (HRs) for overall survival among younger and older patients (per age thresholds in each study).

Results: We identified 103 studies (n = 154,152 patients) that reported outcomes in younger *vs*. older patients with HCC. Younger patients were more likely to undergo curative treatment (RR 1.48, 95% CI 1.24–1.77; I^2 = 99%), although few studies reported treatment among those with early-stage HCC. Younger patients had better survival than older patients (HR 0.87, 95% CI 0.83–0.92; I^2 = 89%), which was consistent in subgroups using age thresholds of <70 years (HR 0.94, 95% CI 0.89–0.99; I^2 = 78%) and <75 years (HR 0.83, 95% CI 0.70–0.98; I^2 = 79%). Younger patients also had better survival in studies of patients with early-stage HCC (HR 0.78, 95% CI 0.65–0.94; I^2 = 60%) and those undergoing curative therapy (HR 0.87, 95% CI 0.77–0.98; I^2 = 87%).

Conclusions: Older patients with HCC are less likely to receive curative treatment and have worse survival than their younger counterparts. Studies to identify factors associated with worse prognosis can inform intervention targets.

© 2025 The Author(s). Published by Elsevier B.V. on behalf of European Association for the Study of the Liver (EASL). This is an open access article under the CC BY license (http://creativecommons.org/licenses/by/4.0/).

Introduction

Hepatocellular carcinoma (HCC) is a leading cause of cancer-related mortality in patients with cirrhosis, with a 5-year survival below 25%.[1,2] The strongest drivers of HCC prognosis are tumor stage at presentation and receipt of curative therapy.[3] Patients with early-stage HCC who undergo surgical procedures achieve a 5-year survival rate surpassing 70%.[4] This survival is in stark contrast to those with more advanced tumors receiving palliative therapies, who experience a 5-year survival below 30%.[4]

The age-dependent nature of HCC risk is well established, with HCC incidence peaking after age 60 years in Western countries.[3] The increasing life expectancy of the population, including patients with cirrhosis, is projected to result in increased numbers of older individuals diagnosed with HCC.[4] Consequently, understanding the presentation and management of HCC in this patient population is paramount.

Effective management of older patients with cancer poses substantial challenges primarily because of heightened susceptibility to concurrent comorbid conditions, which can hinder access to appropriate treatments.[5] Studies from colorectal and lung cancers suggest that older patients are often less likely to receive treatment compared with younger counterparts with similar tumor burden.[6,7] Similarly, older patients with HCC may be less likely to receive treatment than younger counterparts. Furthermore, data on the efficacy and tolerability of cancer treatments in elderly patients is often limited, as this group is underrepresented in clinical trials.[8]

Compared with other cancers in which age and comorbidity have been incorporated into "stopping rules" for cancer screening, HCC surveillance is recommended in patients with cirrhosis or chronic HBV infection without established stopping rules based on age.[9,10] However, a recent modeling study suggested that HCC surveillance may not be cost-effective in patients older than 70 years after hepatitis C cure.[11] This recommendation was partly based on competing risk of mortality and presumed lower eligibility for curative treatments.

* Corresponding author. Address: Division of Digestive and Liver Diseases, University of Texas Southwestern, 5959 Harry Hines Blvd, POB 1, Suite 420, Dallas TX 75390-8887, USA. Tel.: +1 214 645 6029; fax: +1 214 645 6294.
E-mail address: amit.singal@utsouthwestern.edu (A.G. Singal).
† These authors contributed equally and are co-first authors.
https://doi.org/10.1016/j.jhepr.2025.101368

Understanding outcomes among older adults is important to guide recommendations for optimizing HCC management strategies. However, no studies have comprehensively reviewed age-based differences in treatment receipt and clinical outcomes in patients with HCC. Therefore, we conducted a systematic review and meta-analysis to better understand differences in presentation, treatment receipt, and overall survival between younger and older patients with HCC.

Materials and methods

This systematic review was conducted in accordance with the Preferred Reporting Items for Systematic Reviews and Meta-Analyses (PRISMA) reporting guidelines.[12]

Search strategy

We performed an electronic-based search of the PubMed, Ovid MEDLINE, and EMBASE databases to identify all relevant articles and abstracts evaluating studies reporting tumor stage, curative treatment receipt, and/or overall survival among patients with HCC, stratified by age (younger *vs*. older) published between January 2000 and July 2022. The search terms included the following: (liver ca* OR HCC OR hepatocellular ca*) AND (elderly OR old* OR young*). Manual searches of reference lists were also conducted to identify citations that may have been missed by the electronic-based search. Only English articles were considered for further analysis.

Study selection and inclusion/exclusion criteria

Following removal of duplicate citations and the application of inclusion/exclusion criteria, one investigator (OB) screened titles, abstracts, and full texts of the remaining citations to compile a list of potentially relevant articles. A second investigator independently reviewed full texts of included articles for eligibility confirmation and data abstraction, with a third investigator available to resolve disagreements. To mitigate the possibility of missing studies with age-stratified outcomes as a secondary analysis, we used lenient title and abstract screening criteria, with a greater number of studies undergoing full-text review.

Eligible studies encompassed randomized controlled trials and observational studies involving HCC patients aged 18 years or older. We excluded studies that (1) lacked a comparison between younger and older HCC patients, (2) did not include data on treatment receipt or overall survival, (3) were not available in the English language, (4) involved nonhuman data, or (5) were comprised of case reports, review articles, or editorials. In cases of duplicate publications featuring the same patient cohort, the study with the most complete data or most recent was included.

Data extraction and quality assessment

Two investigators (OB and DD) independently extracted data from eligible full texts, with a third investigator (AGS) available to resolve discrepancies. Data elements included author, publication year, country, study design, number of patients, age thresholds defining younger *vs*. older, follow-up duration, proportion of early-stage HCC, type of HCC treatments, and survival estimates. Study quality and bias risk were evaluated using a modified National Institutes of Health Study Quality Assessment Tool.[13]

Exposure of interest

The definition of "younger" *vs*. "older" was determined based on the age threshold specified in each study. In cases where studies reported multiple age thresholds, we used thresholds of either 65 or 70 years for the primary analyses, as these were most commonly reported across studies. Sensitivity analyses were conducted for other reported age thresholds.

Outcome measures

Clinical outcomes of interest included early-stage HCC, curative treatment receipt, and overall survival. For early-stage HCC, we captured the proportion of patients detected at an early stage using the definition of early-stage HCC in each study. If multiple definitions were provided, we preferentially used the Milan criteria or Barcelona Clinic Liver Cancer (BCLC) stage.[14,15] All HCC treatments were recorded, including liver transplantation, surgical resection, local ablation therapy, transarterial therapy (transarterial chemoembolization or transarterial radioembolization), stereotactic body radiation therapy, and systemic therapy; curative treatments was defined as liver transplantation, surgical resection, or local ablation.

Statistical analysis

For each study, we computed the proportions of early-stage HCC and receipt of curative therapy in younger *vs*. older patients. We then calculated pooled risk ratios (RRs) accompanied by 95% CIs for both outcomes using the DerSimonian and Laird method for a random-effects model. We used the χ^2 test of heterogeneity and consistency index (I^2) to quantitatively determine the extent of heterogeneity between studies. I^2 values >75% indicate a high level of heterogeneity, whereas I^2 values between 50% and 75% indicate moderate heterogeneity. We then conducted subgroup analyses to investigate potential sources of heterogeneity. These analyses were preplanned for the following categories: (1) geographic region (Asia *vs*. Europe *vs*. USA *vs*. other), (2) publication year (before 2005, 2005–2009, 2010 –2014, and 2015–2022), (3) type of curative treatment, and (4) age threshold. To assess potential publication bias, we used both visual examination of funnel plots and statistical analysis using Egger's test.[16]

To compare early-stage HCC presentation and treatment receipt between younger and older patients, we calculated a pooled RR. To compare mortality between younger and older patients, we calculated pooled hazard ratios (HRs). For studies that reported HR for survival without a corresponding 95% CI, we computed the 95% CI using the effect estimate and *p* value. For studies reporting more than two HR values, we used the reference age group as the age threshold and conducted pooled analyses of HR values among older patients. Finally, for studies that did not report HR but included 5-year survival data, we calculated the odds ratio (OR) for 5-year survival between younger and older patients. All analyses were conducted using R Studio version 4.2.1 (R Foundation for Statistical Analysis, Vienna, Austria).

Results

Study characteristics

Our literature search identified 14,265 relevant titles published from January 2000 to July 2022. Following screening of titles, 1009 abstracts were reviewed. After a full-text review, 103 studies met the inclusion criteria (Fig. S1); characteristics of these studies are detailed in Tables S1 and S2. Among 154,152 total patients, 101,597 (65.9%) were categorized as "younger" and 52,555 (34.1%) as "older" based on study-reported thresholds. Age thresholds varied across studies, with the most common being 70 years (n = 39) and 75 years (n = 25).

The studies were geographically diverse with 32 conducted in Japan, 18 in China, and 20 in other Asian countries; 15 studies were conducted in Europe, 13 in the US, and the remaining studies elsewhere. Seven studies were published before 2005, 14 between 2005 and 2009, 36 studies 2010 and 2014, and 46 after 2014. There were 26 "all-comer" studies that included patients regardless of tumor stage and treatment receipt, whereas the other studies included focused cohorts by tumor stage or treatment type. Among the 50,931 patients in the "all-comer" studies with receipt of any curative treatment, 32,370 were categorized as "younger" and 18,561 as "older."

Early-stage presentation

Among 21 studies examining tumor stage at presentation (n = 17,813 patients), no difference in early-stage HCC was observed between younger and older patients (RR 1.00, 95% CI 0.94–1.07; I^2 = 35%) (Fig. S2). Early-stage HCC was detected in 36.3% of younger patients *vs*. 35.0% of older patients. Similarly, among the subset of 13 studies that defined "early stage" using BCLC staging or Milan criteria, there was no significant difference in early-stage presentation between younger and older patients (RR 1.02, 95% CI 0.96–1.08; I^2 = 15%). In subgroup analyses, no significant differences were noted in early-stage presentation using age thresholds of 65, 70, and 75 years. Results remained consistent across other subgroups, including geographic location and study period, although there were greater improvements in early-stage detection over time among younger patients than among older patients (Table S3).

Treatment receipt

Among 23 studies reporting curative treatment receipt (n = 42,340 patients), younger patients were more likely to receive curative treatment than older patients (RR 1.48, 95% CI 1.24–1.77) (Fig. 1). Younger patients had curative treatment receipt in 50.4% (40.0–60.6%) of cases compared with older patients at 38.0% (27.6–48.4%) (Table S3). Pooled results were limited by high heterogeneity (I^2 = 99%), which was explored in sensitivity and subgroup analyses. Notable outliers on forest plot inspection were the studies by El-Serag *et al.*[17] and Ozenne *et al.*[18] When these studies were excluded, pooled results remained similar (RR 1.22, 95% CI 1.10–1.37) although heterogeneity persisted (I^2 = 97.4%). When considering various age thresholds defining "older" *vs*. "younger,", there was no difference in curative treatment receipt at thresholds of 65 or 70 years; however, significant differences were noted at a

Study	Experimental Events	Experimental Total	Control Events	Control Total	RR	95% CI	Weight
Liu, Po−Hong, 2014	1,066	2,270	276	812	1.38	[1.24; 1.54]	5.0%
Yan H *et al.*, 2022	12	207	43	1,395	1.88	[1.01; 3.51]	3.1%
Seo *et al.*, 2019	56	346	19	204	1.74	[1.06; 2.84]	3.7%
Oweira *et al.*, 2017	1,876	4,563	581	2,130	1.51	[1.39; 1.63]	5.0%
Borzio *et al.*, 2017	238	527	238	542	1.03	[0.90; 1.18]	4.9%
Yan M *et al.*, 2017	8,447	12,096	2,659	3,743	0.98	[0.96; 1.01]	5.0%
Lee Sang H, 2011	78	149	55	113	1.08	[0.84; 1.37]	4.6%
Tseng Po−Lin *et al.*, 2010	19	55	7	33	1.63	[0.77; 3.45]	2.7%
Mirici−Cappa Federica *et al.*, 2009	372	1,104	238	614	0.87	[0.76; 0.99]	4.9%
Chang Pik−Eu *et al.*, 2008	17	55	101	583	1.78	[1.16; 2.75]	3.9%
Pignata Sandro *et al.*, 2006	219	492	49	158	1.44	[1.11; 1.85]	4.6%
Tsukioka G *et al.*, 2006	90	234	18	91	1.94	[1.25; 3.03]	3.8%
El−serag *et al.*, 2006	268	270	124	2,693	21.56	[18.15; 25.61]	4.8%
Dohmen K *et al.*, 2004	427	668	22	36	1.05	[0.80; 1.37]	4.5%
Lam C−M *et al.*, 2004	40	119	483	1,725	1.20	[0.92; 1.56]	4.5%
Giannini *et al.*, 2002	11	24	26	51	0.90	[0.54; 1.50]	3.6%
Guo Hui *et al.*, 2017	947	1,212	211	318	1.18	[1.08; 1.28]	5.0%
Fan C *et al.*, 2013	160	531	29	164	1.70	[1.20; 2.43]	4.2%
Kim Yun Jung *et al.*, 2012	813	813	179	179	1.00	[0.99; 1.01]	5.0%
Kozyreva O *et al.*, 2011	175	240	55	95	1.26	[1.04; 1.52]	4.8%
Ratana−Amornpin S *et al.*, 2021	36	87	33	97	1.22	[0.84; 1.77]	4.1%
Ozenne *et al.*, 2011	25	40	10	40	2.50	[1.39; 4.50]	3.3%
Hori *et al.*, 2014	200	282	88	140	1.13	[0.97; 1.31]	4.9%
**Random effects model**		**26,384**		**15,956**	**1.48**	**[1.24; 1.77]**	**100.0%**
Heterogeneity: I^2 = 99%, τ^2 = 0.1649, p = 0							

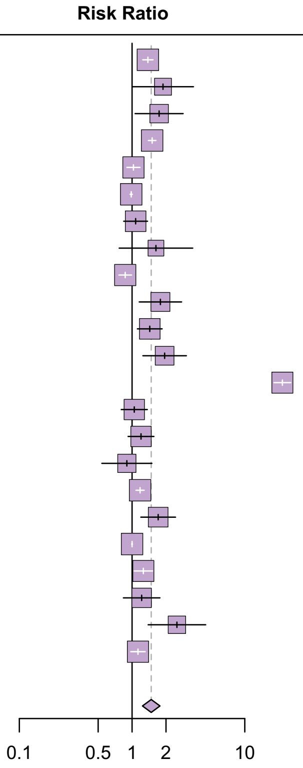

Fig. 1. Receipt of any curative treatment between younger and older patients. Younger patients were more likely to receive curative treatment than older patients (RR 1.48, 95% CI 1.24–1.77). RR, risk ratio.

threshold of 75 years (RR 1.37, 95% CI 1.07–1.75; I^2 = 80%). Across geographic regions, significant differences were not observed in studies from Asia or Europe, although younger patients were more likely to undergo curative treatment than older patients among studies from the USA (RR 2.51, 95% CI 1.02–6.18; I^2 = 100%). Although there was no significant difference in curative treatment receipt between younger and older patients among studies published before 2015, curative treatment was more likely in younger patients among studies published in 2015 or later (RR 1.25, 95% CI 1.02–1.52; I^2 = 96%) (Table S3). Studies attributed differences in curative treatment receipt to several factors including liver dysfunction, performance status, and comorbidity burden (Table S4).

Only three studies compared curative treatment receipt between younger and older patients with early-stage HCC. Guo *et al.*[19] found that older and younger patients with BCLC stage 0-A HCC were equally likely to receive curative therapy (65% *vs*. 65%). Liu *et al.*[20] found that elderly patients were more frequently treated with local ablation than surgical resection compared with younger patients owing to preoperative risk assessment. Conversely, Oweira *et al.*[21] evaluated 6693 patients ≥70 years with early-stage (T1/T2) HCC in the SEER registry and found those aged 70–80 years were more likely to receive surgical or local ablative therapy than those aged >80 years (41.1% *vs*. 27.3%).

Overall survival

A total of 50 studies (n = 123,293) examined HCC survival with outcomes stratified by age. Compared with older patients, younger patients had better survival (HR 0.87, 95% CI 0.83–0.92), although there was a high level of heterogeneity (I^2 = 89%) (Fig. S3). Absolute survival estimates among younger and older patients are described in Table S5**.** When outliers on visual inspection of the forest plots were excluded, younger patients continued to have better survival (HR 0.87, 95% CI 0.83–0.92), and heterogeneity remained high (I^2 = 89%). When considering various age thresholds to define "younger" *vs*. "older" patients, there were significant differences in survival at thresholds of 70 years (HR 0.94, 95% CI 0.89–0.99; I^2 = 78%) and 75 years (HR 0.83, 95% CI 0.70–0.98; I^2 = 79%) but not at lower thresholds of 60 or 65 years. Improved survival in young patients was reported in studies across geographic locations including the USA (HR 0.88, 95% CI 0.78–0.99; I^2 = 94%), Europe (HR 0.80, 95% CI 0.66–0.98; I^2 = 88%), and Asia (HR 0.91, 0.84–0.99; I^2 = 78%). There was no difference in survival among studies published between 2005 and 2009 (HR 1.02, 95% CI 0.79–1.31; I^2 = 85%); however, younger patients had better survival in studies published between 2010 and 2014 (HR 0.89, 95% CI 0.84–0.95; I^2 = 83%) and in 2015 or later (HR 0.88, 95% CI 0.81–0.96; I^2 = 80%).

Among the 14 all-comer studies (n = 33,699) with sufficient data to calculate a pooled estimate, younger patients had better survival (HR 0.88, 95% CI 0.80–0.97; I^2 = 75%) (Fig. 2). When outliers on visual inspection of the forest plots (three with lower hazards and two with higher hazards of mortality) were excluded, younger patients continued to have better survival (HR 0.90, 95% CI 0.83–0.98), and heterogeneity was reduced, albeit still moderate (I^2 = 66%). Across age thresholds, a significant difference in survival was observed at a threshold of 70 years (HR 0.81, 95% CI 0.69–0.96; I^2 = 84%) but not at age 65 years. Survival did not significantly differ by age among studies conducted in Asia (HR 0.90, 95% CI 0.80–1.01; I^2 = 67%) or the USA (HR 0.97, 95% CI 0.75–1.24; I^2 = 66%); however, improved survival in younger patients was reported in the two studies from Europe (HR 0.77, 95% CI 0.61–0.97; I^2 = 72%). Finally, survival did not significantly differ between younger and older patients among studies published between 2005 and 2009 (HR 1.02, 95% CI 0.79–1.31; I^2 = 85%), although differences were observed in studies published between 2010 and 2014 (HR 0.80, 95% CI 0.68–0.94; I^2 = 0%) and those published in 2015 or later (HR 0.87, 95% CI 0.81–0.94; I^2 = 19%).

Few studies distinguished liver *vs*. non-liver mortality between younger and older patients, with most simply reporting the proportion of liver-related *vs*. non-liver-related deaths in

Author	logHR	SE(logHR)	HR	95% CI	Weight
Lee *et al.*, 2022	−0.11	0.0734	0.90	[0.78; 1.04]	10.1%
Seo *et al.*, 2019	−0.49	0.2034	0.61	[0.41; 0.91]	3.9%
Yan M *et al.*, 2017	−0.06	0.0648	0.94	[0.83; 1.07]	10.6%
Lee Sang H, 2011	−0.09	0.1768	0.91	[0.64; 1.29]	4.7%
Tseng Po−Lin *et al.*, 2010	−0.65	0.3315	0.52	[0.27; 1.00]	1.8%
Chang Pik−Eu *et al.*, 2008	0.29	0.1605	1.33	[0.97; 1.82]	5.3%
Pignata Sandro *et al.*, 2006	−0.40	0.1097	0.67	[0.54; 0.83]	7.8%
El−serag *et al.*, 2006	0.28	0.1700	1.32	[0.95; 1.84]	4.9%
Yan Huiwen *et al.*, 2022	−0.30	0.1474	0.74	[0.55; 0.99]	5.8%
Borzio M *et al.*, 2016	−0.16	0.0630	0.85	[0.75; 0.96]	10.8%
Guo Hui *et al.*, 2017	−0.09	0.0900	0.91	[0.76; 1.09]	9.0%
Fan C *et al.*, 2013	−0.19	0.1248	0.83	[0.65; 1.06]	6.9%
Kozyreva O *et al.*, 2011	−0.29	0.1601	0.75	[0.55; 1.03]	5.3%
Yau Thomas *et al.*, 2009	0.01	0.0025	1.01	[1.01; 1.01]	13.3%
**Random effects model**			**0.88**	**[0.80; 0.97]**	**100.0%**

Heterogeneity: I^2 = 75%, τ^2 = 0.0170, p <0.01

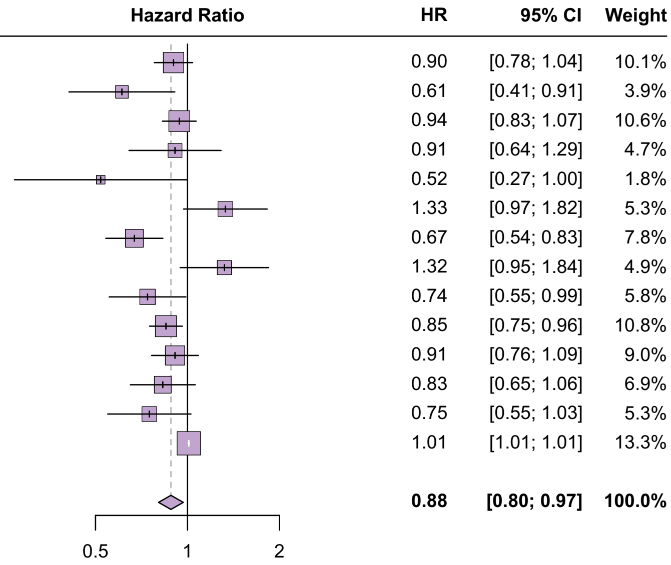

Fig. 2. Overall survival between younger and older patients. Younger patients had overall better survival compared with older patients (HR 0.88, 95% CI 0.80–0.97). HR, hazard ratio.

both subgroups (Table S6). Two studies reported outcomes among patients undergoing a broader range of treatments. Kozyreva *et al.*[22] provided liver-related morality rates for younger and older patients, including median survivals of 24.5 *vs*. 27.8 months and 1-year survival of 66.7% and 66.5%, respectively; however, higher non-liver mortality was observed among older individuals. Conversely, Lee *et al.*[23] found similar liver and non-liver mortality between younger and older individuals. Other studies reported liver and non-liver mortality among specific subgroups of patients, such as those undergoing surgical resection or liver transplantation. Most studies found higher non-liver mortality among older individuals; however, studies were discordant about differential liver-related mortality, with some reporting higher liver mortality among older individuals and others reporting similar mortality.

Of the all-comer studies, only three (n = 922) reported data comparing liver-related *vs*. non-liver-related mortality in younger and older patients. Liver-related mortality did not significantly differ between the two age groups (RR 1.04, 95% CI 0.83–1.31; I^2 = 64%), with liver-related mortality in 52% (95% CI 48.3–55.7%) of older patients and 50% (95% CI 41.9–58.2%) of younger patients (Fig. S5). Conversely, non–liver-related deaths were higher among older patients (25.5%, 95% CI 3.5–54.6%) than among younger patients (5.0%, 95% CI 1.6–8.3%), with a relative risk of 3.36 (95% CI 0.76–14,7; I^2 = 90%) (Fig. S5).

Overall survival among patients with early-stage HCC

Nine studies compared survival among patients with early-stage HCC (Table S7). In four studies with data to calculate a pooled estimate, younger patients had better survival than older patients (HR 0.78, 95% CI 0.65–0.94; I^2 = 60%) (Fig. 3). When Tseng *et al.*[24] was excluded as an outlier study, younger patients continued to have better survival (HR 0.81, 95% CI 0.74–0.88; I^2 = 10%) and heterogeneity was minimal (I^2 = 10%). Of the five studies that compared survival outcomes among older and younger patients with early-stage HCC but did not report HRs (Table 1), three found that younger patients had significantly better survival compared with older patients and two found numerically higher but not significantly improved survival in younger patients. The study by Tsukioka *et al.*[26] found that younger patients with TNM stage I/II had a 5-year survival of ~38% compared with 0% for older patients (≥80 years of age). Borzio *et al.*[25] reported that younger patients with BCLC 0-A had a median survival of >60 months compared with 44 months for older patients (>70 years of age). Guo *et al.* found that younger patients had higher overall survival (37 *vs*. 44 months; p >0.05).

Overall survival among patients after curative treatment

Finally, 63 studies compared survival among those who underwent curative therapy (49 with surgical resection, seven orthotopic liver transplantation, and seven radiofrequency ablation) (Table S2). In the 22 studies with data to calculate a pooled HR, younger patients had better survival than older patients (HR 0.87, 95% CI 0.77–0.98; I^2 = 87%) (Fig. 4). After the exclusion of outliers visualized on the forest plot (two with lower hazards and three with higher hazards), younger patients continued to have better survival (HR 0.86, 95% CI 0.76–0.96; I^2 = 87%). There was no difference in survival between younger and older patients who underwent surgical resection or local ablative therapies; however, younger patients who underwent liver transplantation had improved survival compared with older patients (HR 0.71, 95% CI 0.64–0.78; I^2 = 39%). Differences in survival were greater in magnitude among studies before 2010 but did not reach statistical significance among studies between 2010 and 2014 (HR 0.79, 95% CI 0.60–1.03; I^2 = 78%) or after 2015 (HR 0.91, 95% CI 0.79–1.04; I^2 = 89%). Across age thresholds, a significant difference in survival was observed at a cuff-off of 65 years (HR 0.75, 95% CI 0.61–0.92; I^2 = 78%), but not at higher thresholds of 70 or 75 years.

Sixty-two studies provided data on 5-year survival, among which younger patients had higher 5-year survival (OR 1.21, 95% CI 1.10–1.32; I^2 = 75%) (Fig. S4). Subgroup analysis by type of curative treatment showed improved survival among younger patients receiving surgical resection (OR 1.15, 95% CI 1.03–1.28; I^2 = 67) and local ablative therapies (OR 1.48, 95% CI 1.15–1.91; I^2 = 54%), but differences did not reach significance among those undergoing liver transplantation (OR 1.31, 95% CI 0.999–1.71; I^2 = 92%).

Quality assessment

We found no evidence of publication bias for early-stage HCC presentation (p = 0.05), although there was potential publication bias for studies examining curative treatment (p = 0.04) and overall survival (p = 0.008). Examination of funnel plots suggested a dearth of studies with smaller sample sizes, particularly small negative studies on curative treatment and overall survival differences.

Regarding quality assessment, most studies had clearly defined eligibility criteria and objective outcomes of interest. The most notable limitation of the existent literature was a risk of residual confounding, with only 24 studies matching for important variables including tumor burden, degree of liver dysfunction, and comorbidity. There were an additional 67 studies that adjusted for some but not all confounders. Notably, many studies failed to differentiate liver-related *vs*. non-liver-

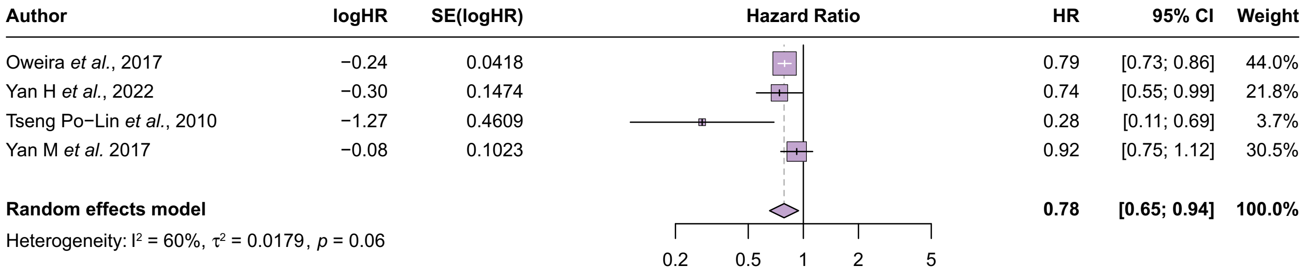

Fig. 3. Overall survival between younger and older patients with early-stage HCC. Younger patients with early-stage HCC had overall better survival compared with older patients (HR 0.78, 95% CI 0.65–0.94). HCC, hepatocellular carcinoma; HR, hazard ratio.

Table 1. Studies reporting overall survival in patients with early-stage HCC.

Study, year	Country	Age cut-off (years)	Number of patients	Staging system	Overall survival in younger *vs.* older, months (95% CI)
Lee 2011[23]	South Korea	<65	<65, n = 149	BCLC A	57.3 (52.2–62.4) *vs.* 53.6 (46.3–60.8), *p* = 0.26
		≥65	≥65, n = 113	TNM I	56.9 (48.6–65.1) *vs.* 45.2 (36.5–54.0), *p* = 0.09
Borzio 2016[25]	Italy	≤70	≤70, n = 527	BCLC 0-A	>60.0 *vs.* 44.0, *p* <0.001
		>70	>70, n = 542		
Guo 2017[19]	China	<65	<65, n = 1,212	BCLC 0-A	44.0 *vs.* 37.0, *p* = 0.05
		≥65	≥65, n = 318		
Tsukioka 2006[26]	Japan	≥80	≥80, n = 91	TNM I	120.0 *vs.* 50.0*, *p* = 0.005
		50–60	50–60, n = 34	TNM II	
Kim 2012[27]	South Korea	<70	<70, n = 813	BCLC 0	76.4 (68.9–83.8) *vs.* 63.0 (45.8–80.3), *p* = 0.36
		≥70	≥70, n = 179	BCLC A	68.2 (60.6–75.8) *vs.* 72.5 (56.6–88.4), *p* = 0.73
				TNM I	68.6 (58.9–78.2) *vs.* 70.9 (53.1–88.6), *p* = 0.55

*Derived from Kaplan Meier curves in Fig. 1 of article. BCLC, Barcelona Clinic Liver Cancer; TNM, tumor node metastasis.

Author	logHR	SE(logHR)	HR	95% CI	Weight
Kao Wei−Yu *et al.*, 2012	−0.89	0.3691	0.41	[0.20; 0.85]	1.9%
Zeng Jian *et al.*, 2020	0.10	0.0486	1.10	[1.00; 1.21]	6.5%
Fujiwara *et al.*, 2014	−0.39	0.0959	0.68	[0.56; 0.82]	5.8%
Pu *et al.*, 2022	−0.02	0.1639	0.98	[0.71; 1.35]	4.5%
Xing *et al.*, 2020	0.30	0.1117	1.35	[1.08; 1.68]	5.5%
Kaibori *et al.*, 2019	−0.20	0.0653	0.82	[0.72; 0.93]	6.3%
Hsu *et al.*, 2018	−0.19	0.1833	0.83	[0.58; 1.19]	4.2%
Doi K *et al.*, 2015	−0.11	0.1988	0.90	[0.61; 1.33]	3.9%
Motoyama H *et al.*, 2014	−0.46	0.1293	0.63	[0.49; 0.81]	5.2%
Hiroki Nishi *et al.*, 2012	0.44	0.2129	1.55	[1.02; 2.35]	3.7%
Tan LY *et al.*, 2021	−0.21	0.0506	0.81	[0.73; 0.89]	6.5%
Diao Yong *et al.*, 2021	0.09	0.0882	1.09	[0.92; 1.30]	5.9%
Adeniji N *et al.*, 2020	−0.26	0.0781	0.77	[0.66; 0.90]	6.1%
Tsuchiya K *et al.*, 2020	0.63	0.4599	1.87	[0.76; 4.61]	1.4%
Chen Zheng *et al.*, 2020	0.04	0.1372	1.04	[0.79; 1.36]	5.0%
Santambrogio *et al.*, 2016	−0.73	0.2338	0.48	[0.30; 0.76]	3.4%
Ha Sang *et al.*, 2015	−0.00	0.2327	1.00	[0.63; 1.57]	3.4%
Kishida N, 2015	−0.13	0.2951	0.88	[0.49; 1.57]	2.6%
Nanashima A *et al.* 2011	1.58	0.6344	4.86	[1.40; 16.85]	0.8%
Kim Joseph *et al.*, 2013	−0.24	0.1087	0.79	[0.64; 0.98]	5.6%
Schwartz *et al.*, 2012	−0.48	0.1475	0.62	[0.46; 0.83]	4.9%
Cullaro *et al.*, 2020	−0.40	0.0305	0.67	[0.63; 0.71]	6.7%
**Random effects model**			**0.87**	**[0.77; 0.98]**	**100.0%**

Heterogeneity: $I^2 = 87\%$, $\tau^2 = 0.0543$, $p < 0.01$

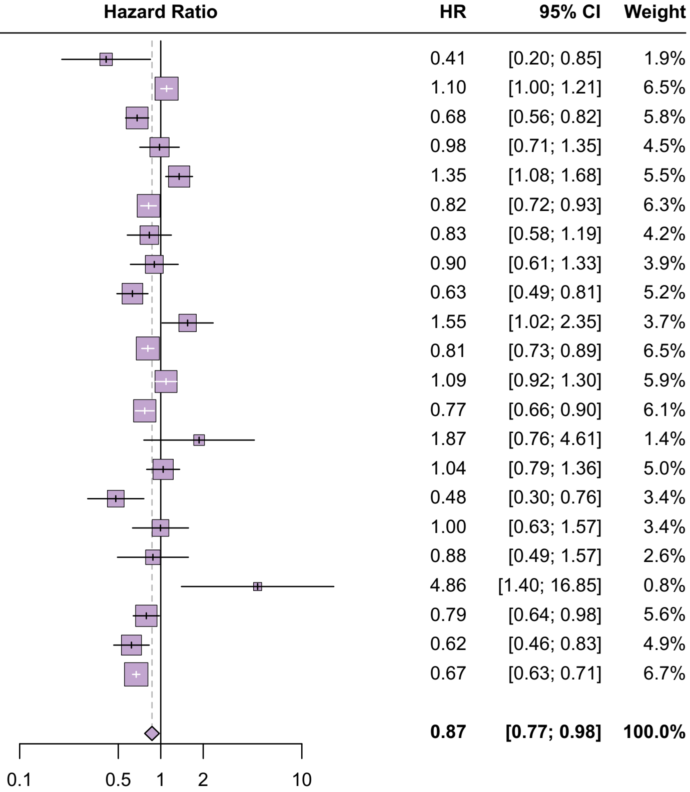

Fig. 4. Overall survival between younger vs. older patients who underwent curative treatment. Younger patients who underwent curative treatment had overall better survival compared with older patients (HR 0.87, 95% CI 0.77–0.98). HR, hazard ratio.

related mortality. Other limitations for most studies were loss to follow-up exceeding 20% at 5 years or loss to follow-up not being reported.

Discussion

Given the increasing number of HCC cases diagnosed in older adults and an aging at-risk population, it is important to understand age-related differences in HCC treatment patterns and prognosis. Despite a similar proportion of younger and older patients having tumors diagnosed at an early stage, we found that younger patients were significantly more likely to undergo curative treatment and exhibit better overall survival. These differences in survival persisted when examining the subset of patients detected at an early stage and the subset that received curative treatment. Age thresholds to define "older" *vs.* "younger" were inconsistent across studies; however, differences in curative therapy and survival appeared greatest in patients ≥75 years. Although there were no significant differences in curative treatment receipt, early-stage HCC detection, or overall survival between younger and older patients during earlier study periods, these differences became more pronounced over time. There appeared to be greater improvements in early-stage presentation and overall survival over time among younger than older patients.

We found no significant differences in early-stage detection between younger and older individuals. Although our included studies did not provide data on HCC surveillance receipt, prior meta-analyses found no significant differences by age.[28] Despite similar proportions of older adults being diagnosed with early-stage HCC, they are less likely to receive curative treatment. The reasons behind this disparity are complex and may be attributed to many possible clinical factors including differences in liver

dysfunction, performance status, or comorbidity burden.[5,29] Alternatively, this disparity may also relate to differences in patient preferences such as decreased willingness to proceed with invasive surgical therapies, differences in social support to engage in healthcare decisions, differences in access (*e.g.* ability to drive to appointments), or implicit physician bias. We found that the greatest disparity in curative treatment receipt was among patients aged ≥75 years, which may be driven by differential access to surgical procedures.[30,31] Although there is no universally accepted age threshold for liver transplantation, older patients may derive fewer life-years gained.[32,33] Interestingly, we found no significant differences in treatment receipt at lower age thresholds, specifically >65 years, suggesting acceptance of surgical therapies by patients and providers for patients aged 65–75 years.

Finally, we found that older patients had significantly worse survival than younger patients overall, likely in part related to lower rates of curative treatment receipt. However, this disparity persisted among the subgroups of patients with early-stage HCC and those who underwent curative treatment. Once again, the factors driving this disparity are likely multifactorial, with potential factors including age-related differences in liver dysfunction, prevalence of comorbid conditions, and overall performance status. Interestingly, the greatest difference was observed in the subset of patients who underwent liver transplantation, despite generally stringent selection criteria aimed at identifying optimal candidates for transplant. Although there are different HCC subtypes and variation in tumor biology, prior studies have not reported more aggressive tumor biology in older patients compared with younger patients.[34,35] Therefore, this survival difference may instead be related to a higher competing risk of non-liver-related mortality in older patients.

Further studies are needed to characterize reasons driving differences in curative treatment to see if this disparity can be mitigated. Conversely, the benefits of early-stage HCC detection may be mitigated in older patients, and these data would inform stopping rules for HCC surveillance programs. Stopping rules are helpful to maximize the overall value of surveillance programs, as older patients may be at greatest risk of surveillance harms but derive fewer benefits.[36–40] Importantly, future studies will need to examine the intersection between age and other factors such as comorbidity burden and liver dysfunction. For example, surveillance is likely to be of greater benefit in an otherwise healthy 75-year-old patient with Child– Pugh A cirrhosis than in a 65-year-old patient with Child–Pugh B cirrhosis and significant comorbidities (*e.g.* congestive heart failure or renal failure). These concepts have been incorporated into other cancer screening programs such as colorectal cancer screening and can similarly be considered for HCC surveillance.[41] The AASLD recommends consideration of comorbidity, performance status, and patient preferences when assessing the value of HCC surveillance in individuals, but further data are needed to codify how to incorporate these factors into decisions.

Our results should be interpreted considering the study's limitations. First, our meta-analysis was conducted at the study level, and we did not have patient-level data for additional analyses to explore observed age disparities. Specifically, this precluded our ability to examine important confounders, including liver dysfunction and comorbidity. Second, several of our pooled analyses had moderate to severe heterogeneity, which we tried to address through subgroup analyses; however, heterogeneity could not be fully resolved in all cases. The heterogeneity is evident with variations in age thresholds, study populations, and definitions of early-stage HCC across studies. Third, there was a suggestion of publication bias with potential under-reporting of small negative studies. Fourth, some studies with relevant age-stratified outcomes may have been missed by our literature search, particularly if not the primary aim of the study; however, we attempted to mitigate this possibility by using lenient title and abstract screening criteria, with a greater number of studies undergoing full-text review. Fifth, studies evaluating survival after curative treatments may be prone to differential selection bias among younger and older patients, and it is unclear if the same findings would hold among broader populations eligible for curative treatment. Finally, the interpretation of the pooled results in our meta-analysis is limited by the quality of the included studies. Many studies had a risk of residual confounding and failed to report loss to follow-up for survival analysis. Further, most studies failed to account for intersectionality between other known differences in HCC treatment and prognosis, including race/ethnicity, socioeconomic status, and rural–urban geography.[42–45] Many studies also did not differentiate liver *vs*. non-liver mortality despite the expected higher non-liver mortality among older adults. These gaps in analyses and reporting should be addressed in future studies examining potential differences in outcomes by age.

Conclusions

There are consistent and persistent age differences in HCC treatment and prognosis, with younger patients more likely to receive curative therapies and have improved overall survival compared with older patients. Studies should examine reasons for these differences and test interventions to mitigate any contributing failures in healthcare delivery processes.

Affiliations

[1]*Department of Internal Medicine, University of Texas Southwestern Medical Center, Dallas, TX, USA;* [2]*Department of Internal Medicine, University of Michigan, Ann Arbor, MI, USA;* [3]*Department of Internal Medicine, University of Chicago, Chicago, IL, USA;* [4]*Department of Internal Medicine, Northwestern University, Evanston, IL, USA;* [5]*Department of Internal Medicine, Cedars-Sinai Medical Center, Los Angeles, CA, USA;* [6]*Department of Hepatology and Liver Transplantation, AIG Hospitals, Hyderabad, India*

Abbreviations

BCLC, Barcelona Clinic Liver Cancer; HCC, hepatocellular carcinoma; HR, hazard ratio; OR, odds ratio; RR, risk ratio.

Financial support

AGS's research is conducted with support from the National Cancer Institute (R01 CA212008, R01 CA222900, and R01 MD012565) and Cancer Prevention Research Institute of Texas (RP200554). DD's research is conducted with support from CPRIT RP210041. The content is solely the responsibility of the authors and does not necessarily represent the official views of the National Institutes of Health, Cancer Prevention Research Institute of Texas, or the United States government.

Conflicts of interest

AGS has served as a consultant or on advisory boards for Genentech, AstraZeneca, Eisai, Exelixis, Bayer, Boston Scientific, Sirtex, Histosonics, FujiFilm Medical Sciences, Exact Sciences, Roche, Glycotest, Freenome, and GRAIL.

NDP has served as a consultant or on advisory boards for Eisai, Exelixis, FujiFilm Medical Sciences, Sirtex, AstraZeneca, and Gilead. NER has served as consultant or on advisory boards for AstraZeneca, Eisai, Exelixis, and Genentech. AP is on the medical advisory board for Genentech, AstraZeneca, and Replimune. JDY provides a consulting service for AstraZeneca, Eisai, Exact Sciences, and FujiFilm Medical Sciences. None of the other authors have any relevant conflicts of interest to disclose.

Please refer to the accompanying ICMJE disclosure forms for further details.

Authors' contributions

Conceptualization: AGS. Data curation: OB, DD. Formal analysis: DD. Writing—original draft: OB, DD, AGS. Writing—review and editing: all authors. Have read and approved the final version of the manuscript for submission: all authors.

Data availability statement

No new data were generated as part of this study. All new analyses are presented in the manuscript.

Supplementary data

Supplementary data to this article can be found online at https://doi.org/10.1016/j.jhepr.2025.101368.

References

Author names in bold designate shared co-first authorship

[1] Rumgay H, Arnold M, Ferlay J, et al. Global burden of primary liver cancer in 2020 and predictions to 2040. J Hepatol 2022;77:1598–1606.
[2] Liu Y, Zheng J, Hao J, et al. Global burden of primary liver cancer by five etiologies and global prediction by 2035 based on global burden of disease study 2019. Cancer Med 2022;11:1310–1323.
[3] Singal AG, Kanwal F, Llovet JM. Global trends in hepatocellular carcinoma epidemiology: implications for screening, prevention and therapy. Nat Rev Clin Oncol 2023;20:864–884.
[4] Singal AG, Kudo M, Bruix J. Breakthroughs in hepatocellular carcinoma therapies. Clin Gastroenterol Hepatol 2023;21:2135–2149.
[5] Hyder O, Dodson RM, Nathan H, et al. Referral patterns and treatment choices for patients with hepatocellular carcinoma: a United States population-based study. J Am Coll Surg 2013;217:896–906.
[6] Pham J, Conron M, Wright G, et al. Excess mortality and undertreatment in elderly lung cancer patients: treatment nihilism in the modern era? ERJ Open Res 2021;7. 00393–2020.
[7] Millan M, Merino S, Caro A, et al. Treatment of colorectal cancer in the elderly. World J Gastrointest Oncol 2015;7:204–220.
[8] Federico P, Giunta EF, Pappalardo A, et al. How to treat hepatocellular carcinoma in elderly patients. Pharmaceuticals (Basel) 2021;14:233.
[9] Singal AG, Llovet JM, Yarchoan M, et al. AASLD Practice Guidance on prevention, diagnosis, and treatment of hepatocellular carcinoma. Hepatology 2023;78:1922–1965.
[10] European Association for the Study of the Liver. EASL Clinical Practice Guidelines: management of hepatocellular carcinoma. J Hepatol 2018;69:182–236.
[11] Mueller PP, Chen Q, Ayer T, et al. Duration and cost-effectiveness of hepatocellular carcinoma surveillance in hepatitis C patients after viral eradication. J Hepatol 2022;77:55–62.
[12] Page MJ, McKenzie JE, Bossuyt PM, et al. The PRISMA 2020 statement: an updated guideline for reporting systematic reviews. BMJ 2021;372:n71.
[13] National Heart, Lung, and Blood Institute. Study Qual Assess tools 2019. https://www.nhlbi.nih.gov/health-topics/study-quality-assessment-tools. [Accessed 1 November 2022].
[14] Mazzaferro V, Regalia E, Doci R, et al. Liver transplantation for the treatment of small hepatocellular carcinomas in patients with cirrhosis. N Engl J Med 1996;334:693–699.
[15] Reig M, Forner A, Rimola J, et al. BCLC strategy for prognosis prediction and treatment recommendation: the 2022 update. J Hepatol 2022;76:681–693.
[16] Egger M, Davey Smith G, Schneider M, et al. Bias in meta-analysis detected by a simple, graphical test. BMJ 1997;315:629–634.
[17] El-Serag HB, Siegel AB, Davila JA, et al. Treatment and outcomes of treating of hepatocellular carcinoma among Medicare recipients in the United States: a population-based study. J Hepatol 2006 Jan;44(1):158–166. https://doi.org/10.1016/j.jhep.2005.10.002. Epub 2005 Nov 2. PMID: 16290309.
[18] Ozenne V, Bouattour M, Goutté N, et al. Prospective evaluation of the management of hepatocellular carcinoma in the elderly. Dig Liver Dis 2011 Dec;43(12):1001–1005. https://doi.org/10.1016/j.dld.2011.06.019. Epub 2011 Jul 27. PMID: 21798829.
[19] Guo H, Wu T, Lu Q, et al. Hepatocellular carcinoma in elderly: Clinical characteristics, treatments and outcomes compared with younger adults. PLoS One 2017;12(9):e0184160. https://doi.org/10.1371/journal.pone.0184160. PMID: 28886106; PMCID: PMC5590882.
[20] Liu PH, Hsu CY, Lee YH, et al. Uncompromised treatment efficacy in elderly patients with hepatocellular carcinoma: a propensity score analysis. Medicine (Baltimore) 2014 Dec;93(28):e264. https://doi.org/10.1097/MD.0000000000000264. Erratum in: Medicine (Baltimore). 2015 Jan;94(4):1. doi: 10.1097/01.md.0000461199.07356.ca. PMID: 25526453; PMCID: PMC4603105.
[21] Oweira H, Petrausch U, Helbling D, et al. Early stage hepatocellular carcinoma in the elderly: A SEER database analysis. J Geriatr Oncol 2017 Jul;8(4):277–283. https://doi.org/10.1016/j.jgo.2017.03.002. Epub 2017 Apr 4. PMID: 28389117.
[22] Kozyreva ON, Chi D, Clark JW, et al. A multicenter retrospective study on clinical characteristics, treatment patterns, and outcome in elderly patients with hepatocellular carcinoma. Oncologist 2011;16(3):310–318. https://doi.org/10.1634/theoncologist.2010-0223. Epub 2011 Feb 24. PMID: 21349948; PMCID: PMC3228108.
[23] Lee SH, Choi HC, Jeong SH, et al. Hepatocellular carcinoma in older adults: clinical features, treatments, and survival. J Am Geriatr Soc 2011 Feb;59(2):241–250. https://doi.org/10.1111/j.1532-5415.2010.03273.x. Epub 2011 Jan 28. PMID: 21275934.
[24] Tseng PL, Wang JH, Tung HD, et al. Optimal treatment increased survival of hepatocellular carcinoma patients detected with community-based screening. J Gastroenterol Hepatol 2010 Aug;25(8):1426–1434. https://doi.org/10.1111/j.1440-1746.2010.06285.x. PMID: 20659234.
[25] Borzio M, Dionigi E, Vitale A, et al. Management and prognosis of hepatocellular carcinoma in the elderly: Results of an in-field multicenter cohort study. Liver Int 2017 Aug;37(8):1184–1192. https://doi.org/10.1111/liv.13392. Epub 2017 Mar 30. PMID: 28214386.
[26] Tsukioka G, Kakizaki S, Sohara N, et al. Hepatocellular carcinoma in extremely elderly patients: an analysis of clinical characteristics, prognosis and patient survival. World J Gastroenterol 2006 Jan 7;12(1):48–53. https://doi.org/10.3748/wjg.v12.i1.48. PMID: 16440416; PMCID: PMC4077478.
[27] Kim YJ, Jang BK, Kim ES, et al. Hepatocellular carcinoma in the elderly: clinical characteristics, treatment, survival analysis in Korean patients older than 70 years. J Korean Med Sci 2012 Oct;27(10):1147–1154. https://doi.org/10.3346/jkms.2012.27.10.1147. Epub 2012 Oct 2. PMID: 23091310; PMCID: PMC3468749.
[28] Wolf E, Rich NE, Marrero JA, et al. Use of hepatocellular carcinoma surveillance in patients with cirrhosis: a systematic review and meta-analysis. Hepatology 2021;73:713–725.
[29] Janssen-Heijnen ML, Houterman S, Lemmens VE, et al. Prognostic impact of increasing age and co-morbidity in cancer patients: a population-based approach. Crit Rev Oncol Hematol 2005;55:231–240.
[30] Mathur AK, Ashby VB, Fuller DS, et al. Variation in access to the liver transplant waiting list in the United States. Transplantation 2014;98:94–99.
[31] Kemmer N, Safdar K, Kaiser TE, et al. Liver transplantation trends for older recipients: regional and ethnic variations. Transplantation 2008;86:104–107.
[32] Schaubel DE, Guidinger MK, Biggins SW, et al. Survival benefit-based deceased-donor liver allocation. Am J Transpl 2009;9:970–981.
[33] Goudsmit BFJ, Ilaria P, Tushuizen ME, et al. Survival benefit from liver transplantation for patients with and without hepatocellular carcinoma. JHEP Rep 2023;5:100907.
[34] Nathani P, Gopal P, Rich N, et al. Hepatocellular carcinoma tumour volume doubling time: a systematic review and meta-analysis. Gut 2021;70:401–407.
[35] Rich NE, John BV, Parikh ND, et al. Hepatocellular carcinoma demonstrates heterogeneous growth patterns in a multicenter cohort of patients with cirrhosis. Hepatology 2020;72:1654–1665.
[36] Atiq O, Tiro J, Yopp AC, et al. An assessment of benefits and harms of hepatocellular carcinoma surveillance in patients with cirrhosis. Hepatology 2017;65:1196–1205.
[37] Narasimman M, Hernaez R, Cerda V, et al. Hepatocellular carcinoma surveillance may be associated with potential psychological harms of in patients with cirrhosis. Hepatology 2024;79:107–117.
[38] Narasimman M, Hernaez R, Cerda V, et al. Financial burden of hepatocellular carcinoma screening in patients with cirrhosis. Clin Gastroenterol Hepatol 2024;22:760–767.e1.

[39] Singal AG, Patibandla S, Obi J, et al. Benefits and harms of hepatocellular carcinoma surveillance in a prospective cohort of patients with cirrhosis. Clin Gastroenterol Hepatol 2021;19:1925–19232 e1.
[40] **Singal AG**, **Zhang E**, Narasimman M, et al. HCC surveillance improves early detection, curative treatment receipt, and survival in patients with cirrhosis: a meta-analysis. J Hepatol 2022;77:128–139.
[41] Issaka RB, Chan AT, Gupta S. AGA clinical practice update on risk stratification for colorectal cancer screening and post-polypectomy surveillance: expert review. Gastroenterology 2023;165:1280–1291.
[42] Rich NE, Carr C, Yopp AC, et al. Racial and ethnic disparities in survival among patients with hepatocellular carcinoma in the United States: a systematic review and meta-analysis. Clin Gastroenterol Hepatol 2022;20:e267–e288.
[43] Rich NE, Murphy CC, Yopp AC, et al. Sex disparities in presentation and prognosis of 1110 patients with hepatocellular carcinoma. Aliment Pharmacol Ther 2020;52:701–709.
[44] Wagle NS, Park S, Washburn D, et al. Racial and ethnic disparities in hepatocellular carcinoma treatment receipt in the United States: a systematic review and meta-analysis. Cancer Epidemiol Biomarkers Prev 2024;33:463–470.
[45] Wagle NS, Park S, Washburn D, et al. Racial, ethnic, and socioeconomic disparities in treatment delay among patients with hepatocellular carcinoma in the United States. Clin Gastroenterol Hepatol 2023;21:1281.

Keywords: Liver cancer; Elderly; Disparities; Prognosis; Treatment.

Received 23 September 2024; received in revised form 14 January 2025; accepted 18 February 2025; Available online 26 February 2025

Journal of Hepatology, Volume *7*

Supplemental information

Impact of age on clinical outcomes among patients with hepatocellular carcinoma: A systematic review and meta-analysis

Olgert Bardhi, Darine Daher, Mausam Patel, Karim Seif El-Dahan, Nicole E. Rich, Sukul Mitta, Neehar D. Parikh, Anjana Pillai, Laura M. Kulik, Ju Dong Yang, Anand V. Kulkarni, Purva Gopal, and Amit G. Singal

Impact of age on clinical outcomes among patients with hepatocellular carcinoma: A systematic review and meta-analysis

Olgert Bardhi, Darine Daher, Mausam Patel, Karim Seif El-Dahan, Nicole E. Rich, Sukul Mitta, Neehar D. Parikh, Anjana Pillai, Laura M. Kulik, Ju Dong Yang, Anand V. Kulkarni, Purva Gopal, Amit G. Singal

Table of contents

Fig. S1………………………………………………………….………………………. 2

Fig. S2……………………………………………………………………………………….3

Fig. S3……………………………………………………………………………………….4

Fig. S4……………………………………………………………………………………….5

Fig. S5……………………………………………………………………………………….6

Table S1……………………………………………………………………………………....7

Table S2……………………..……………………………………………………………..11

Table S3……………………..……………………………………………………………..16

Table S4……………………..……………………………………………………………..17

Table S5……………………..……………………………………………………………..19

Table S6……………………..……………………………………………………………..25

Table S7……………………..……………………………………………………………..26

Fig. S1. Flow Diagram of Study Selection Process

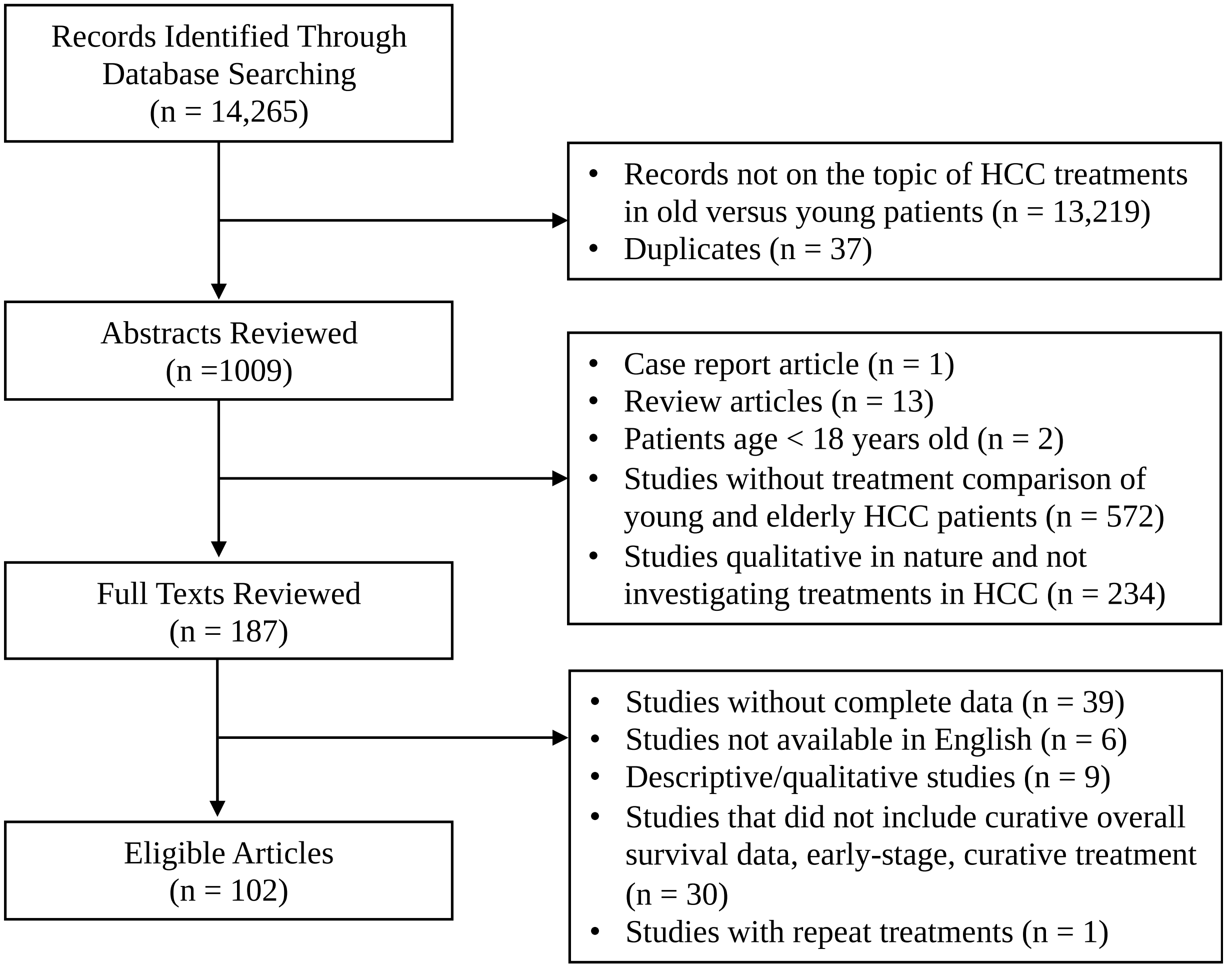

Fig. S2. Early-stage presentation between younger vs. older patients

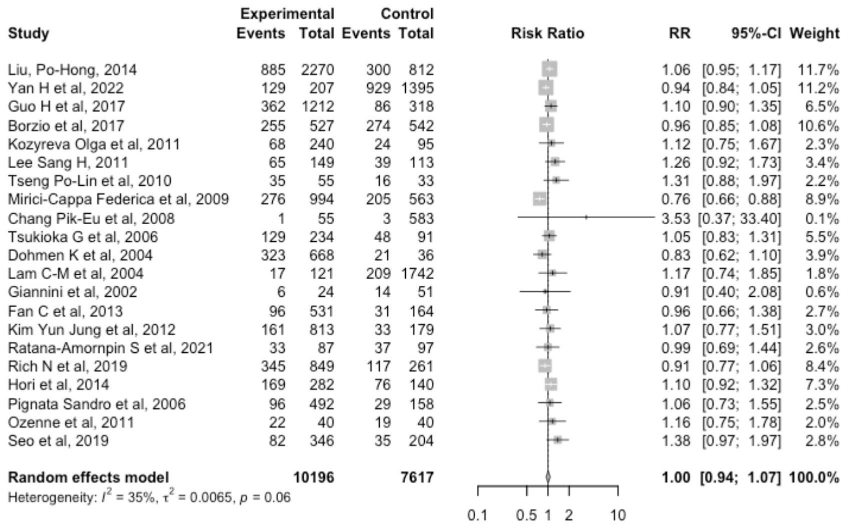

There was no significant difference in early-stage HCC presentation between young versus older patients (RR 1.00; 95%CI 0.94 – 1.07).

Fig. S3. Overall survival between younger vs. older patients

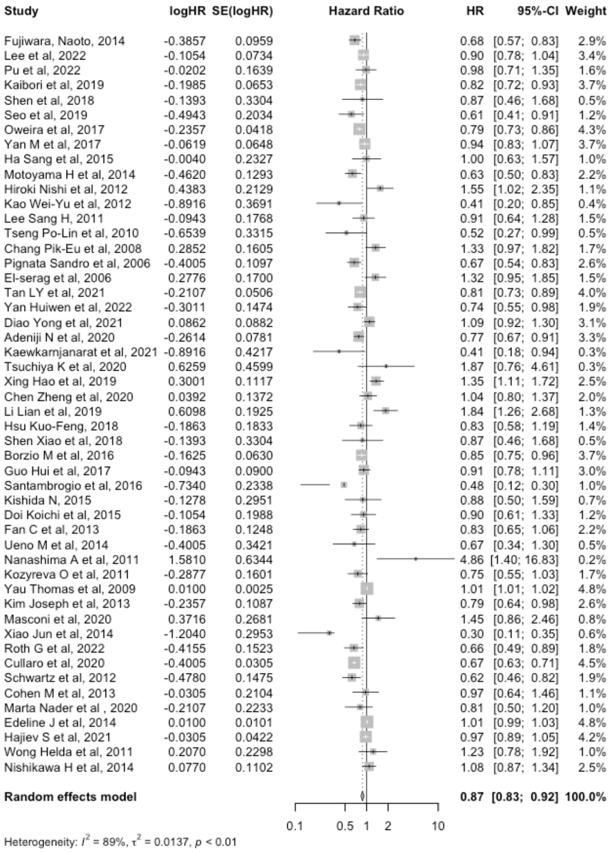

Younger patients had better overall survival than older patients (HR 0.87; 95%CI 0.83 – 0.92).

Fig. S4. Five-year survival between younger vs. older patients who underwent curative treatment.

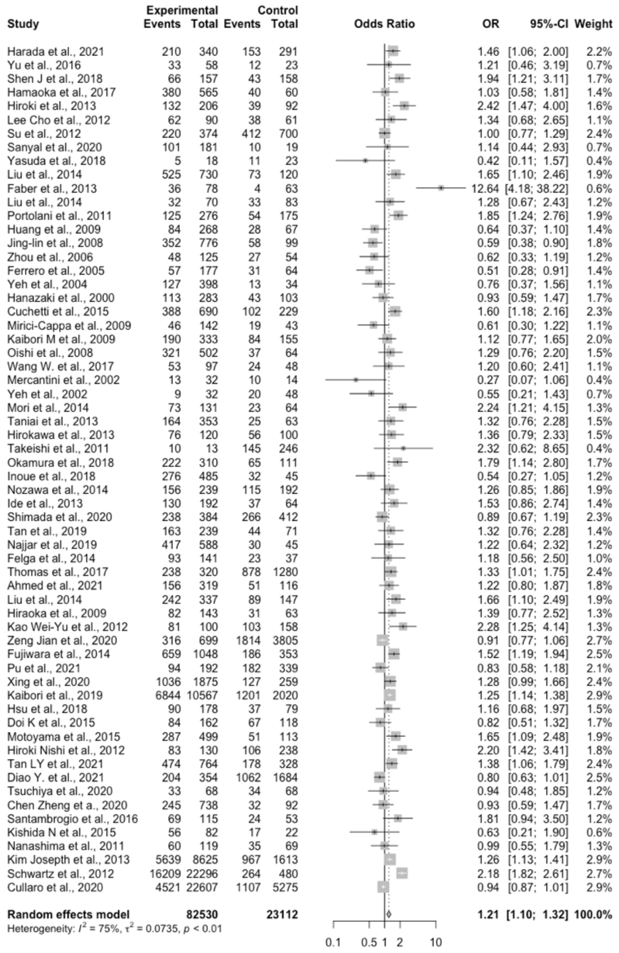

Younger patients who underwent curative treatment showed higher odds of 5-year survival compared to older patients (OR 1.21; 95%CI 1.10 – 1.32).

Fig. S5. Liver and Non-Liver Related Mortality in Younger and Older Patients

Liver Related Mortality

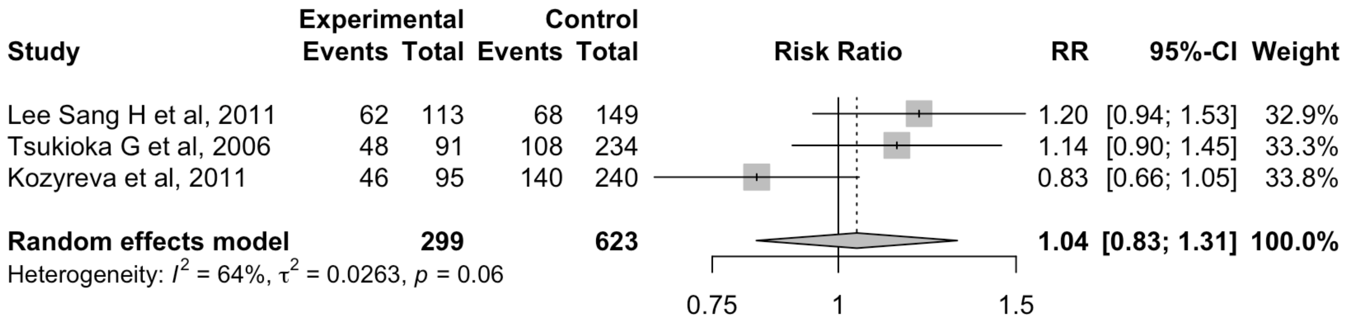

Non-Liver Related Mortality

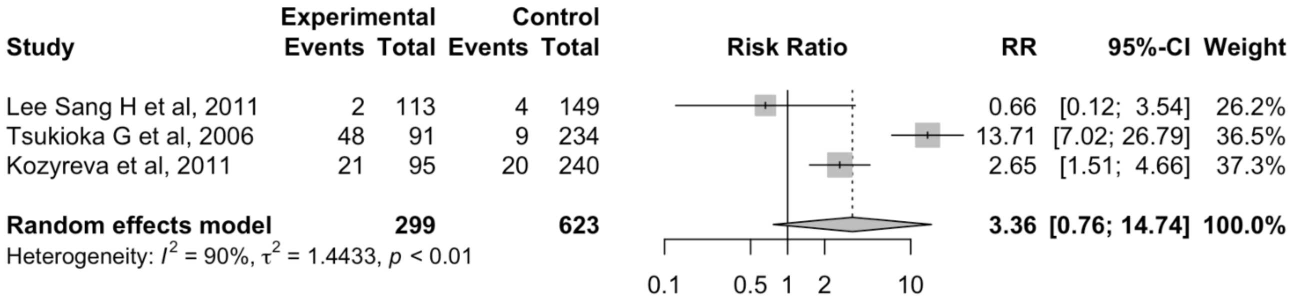

There was no difference in liver related mortality in older and younger patients (RR 1.04, 95%CI 0.83 – 1.31; I^2=64%). However, non–liver-related deaths were higher among older patients than younger patients (RR 3.36 ,95%CI 0.76–14.74; I^2=90%).

Table S1. Characteristics of all-comer studies investigating curative treatments.

Study, Year	Country	Cohort	Treatment	Age Cutoff	Number of Patients	Staging System
**Liu, Po-Hong, 2014**	Taiwan	Elderly: HCV 34%, HBV 36%<br><br>Young: HCV 29%, HBV 61%	Surgical Resection, RFA	< 75<br>≥ 75	3082	BCLC Stage 0 - A
**Yan H et al., 2022**	China	Elderly: HBV 88.2%, HCV 4.9%<br><br>Young: HBV 96.1%, HCV 0.5%	Surgical Resection	≤ 45<br>> 45	1602	BCLC Stage 0 - B
**Seo et al., 2019**	Korea	>Elderly: HBV 3.9%, HCV 37.3%<br><br>Elderly: HBV 15%, HCV 30.1%<br><br>Young: HBV 19.4%, HCV 25.7%	Surgical Resection, RFA	≥ 85<br>80 - 85<br>75 - 80 (cut off)	550	BCLC Stage 0 - A
**Oweira et al., 2017**	USA	N/A	Electrocautery, cryosurgery, laser, PEI, RFA, acetic acid injection.	70 - 80<br>> 80	6693	TNM Stage 1 & 2
**Borzio et al., 2017**	Italy	Elderly: HBV 9.2%, HCV 61.6%<br><br>Young: HBV 18.6%, HCV 49.3%	Surgical Resection, OLT, RFA	> 70<br>≤ 70	1069	BCLC Stage 0 - A
**Yan M et al., 2017**	USA	N/A	Surgical Resection, OLT, PEI, RFA	<50 (reference)<br>50-59<br>60 - 69	48347	Milan

				> 70 (cutoff)		
**Lee Sang H., 2011**	South Korea	Elderly: HBV 45.1%, HCV 23.9%<br><br>Young: HBV 75.8%, HCV 5.4%	Surgical Resection, OLT, RFA, PEI	< 65<br>≥ 65	262	BCLC Stage 0 - A
**Tseng Po-Lin et al., 2010**	Taiwan	Elderly: HCV 69.7%<br><br>Young: HCV 81.8%	Surgical Resection, RFA, PEI	< 70<br>≥ 70	88	BCLC Stage 0 - A
**Mirici-Cappa Federica et al., 2009**	Italy	Elderly: HBV 7.4%, HCV 62.8%<br><br>Young: HBV 11.9%, HCV 47.4%	Surgical Resection, RFA	< 70<br>≥ 70	1718	CLIP
**Chang Pik-Eu et al., 2008**	Singapore	Elderly: HCV 59.7%<br><br>Young: HCV 85.5%	Surgical Resection, RFA	≤ 40<br>> 40	638	TNM Stage 1
**Pignata Sandro et al., 2006**	Italy	Elderly: HBV 13%, HCV 84%<br><br>Young: HBV 23%, HCV 77%	Surgical Resection, PEI	< 70<br>≥ 70	650	CLIP
**Tsukioka G et al, 2006**	Japan	Elderly: HBV 3.2%, HCV 73.6%<br><br>Young: HBV 15.3%, HCV 77.7%	OLT, RFA	≥ 80<br>50 - 60	325	TNM Stage 1 & 2
**El-serag et al, 2006**	USA	N/A	Surgical Resection, RFA, OLT	>65 (cut off)<br>65-74<br>75+	2963	N/A

**Dohmen K et al., 2004**	Japan	Elderly: HBV 2.7%, HCV 83.3%<br><br>Young: HBV 10.1%, HCV 85.9%	Surgical Resection, PEI, RFA	< 80<br>≥ 80	704	N/A
**Lam C-M et al., 2004**	Hong Kong	Elderly: HBV 100%<br>Young: HBV 100%	Surgical Resection	≤ 40<br>> 40	1844	TNM Stage 1 & 2
**Giannini et al., 2002**	Italy	Elderly: HCV 100%<br><br>Young: HCV 100%	Surgical Resection, OLT, PEI, PEI+TACE	< 65<br>≥ 65	75	CLIP
**Guo Hui et al., 2017**	China	Elderly; HBV 49.4%, HCV 15.1%<br><br>Young: HBV 79.7%, HCV 3.2%	Surgical Resection, OLT, RFA, RFA/TACE/PEI	< 65<br>≥ 65	1530	BCLC Stage 0-A
**Fan C et al., 2013**	China	Elderly: HCV 23.8%, HBV 40.9%<br><br>Young: HCV 5.6%, HBV 76.6%	Surgical Resection, RFA	< 70<br>≥ 70	695	BCLC Stage 0 - A
**Kim Yun Jung et al., 2012**	South Korea	Elderly: HCV 26.3%, HBV 31.3%<br><br>Young: HCV 9.2%, HBV 69.2%	Surgical Resection, TACE, RFA/PEIT, Chemotherapy, Palliative	< 70<br>≥ 70	992	BCLC Stage 0 - A
**Kozyreva O et al., 2011**	USA	Elderly: HBV 14.7%, HCV 21.1%<br><br>Young: HBV 21.2%, HCV 48.3%	Surgical Resection, RFA, TACE, Radiation, OLT, Surgical Resection	< 70<br>≥ 70	335	TNM Stage 1

**Ratana-Amornpin S et al., 2021**	Thailand	Elderly: HBV 26.6%, HCV 25.5%<br><br>Young: HBV 54.9%, HCV 28.1%	Surgical Resection, RFA	< 65<br>≥ 65	184	BCLC Stage 0 - A
**Ozenne et al., 2011**	France	Elderly:<br>HBV 12.5%, HCV 57.5%<br><br>Young:<br>HBV 30%, HCV 32.5%	OLT, surgical resection, RFA	> 75<br>< 75	80	BCLC Stage A
**Hori et al., 2014**	Japan	Young:<br>HBV 11.3%, HCV 80.8%<br><br>Elderly:<br>HBV 3.5%, HCV 80.7%	Surgical Resection, RFA, RFA/TACE	< 75<br>≥ 75	422	TNM Stage 1 & 2

Abbreviations: HBV, hepatitis B virus; HCV, hepatitis C virus; RFA, radiofrequency ablation; TACE, transarterial chemoembolization; OLT, orthotopic liver transplantation; PEI, percutaneous ethanol injection; BCLC, Barcelona Clinic Liver Cancer; TNM, tumor node metastasis; CLIP, cancer of the liver Italian program.

Table S2. Studies reporting overall survival among patients who underwent curative treatment

Study, Year	Country	Age Cut-off	Number of Patients	Curative Treatment	Overall Survival		Significance (younger vs. older)
					Younger 1-, 3-, 5-year	Older 1-, 3-, 5-year	
**Harada et al., 2021**	Japan	< 70<br>≥ 70	< 70, n= 340<br>≥ 70, n= 291	Resection	92.8%, 76%, 61.8%	89.1%, 72.9%, 52.9%	**p< 0.01**
**Yu et al., 2016**	China	< 70<br>≥ 70	≤ 70, n= 58<br>> 70, n= 23	Resection	79.1%, 61.1%, 57.1%	82.6%, 73.9%, 56.5%	p= 0.75
**Shen J et al., 2018**	China	≤ 55<br>> 55	≤ 55, n= 157<br>> 55, n= 158	Resection	86.5%, 57.5%, 42.4%	78.1%, 45.1%, 27.4%	**p= 0.007**
**Hamaoka et al., 2017**	Japan	≥80<br><80	≥ 80, n= 60<br>< 80, n=565	Resection	99.5%, 80.3%, 67.4%	92.7%, 77.5%, 67.1%	p= 0.62
**Hiroki et al., 2013**	Japan	≥ 75<br>< 75	≥ 75, n= 92<br>< 75, n= 206	Resection	91.0%, 77.5%, 64.4%	90.0%, 73.3%, 43.0%	p= 0.19
**Lee Cho et al., 2012**	South Korea	< 70<br>≥ 70	≤ 40, n= 90<br>≥ 70, n= 61	Resection	94.4%, 75.1%, 69.1 %	86.5%, 75.1%, 62.3%	NS*
**Su et al., 2012**	Taiwan	≤ 55<br>> 55	≤ 55, n= 374<br>> 55, n= 700	Resection	82.4%, 67.3%, 58.9%	86.7%, 66.4%, 51.4%	**p= 0.02****
**Sanyal et al., 2020**	UK	≥80<br><80	≥ 80, n= 19<br>< 80, n= 181	Resection	83.4%, 65.7%, 56.3%	88.2%, 81.9%, 55.8%	p= 0.83
**Yasuda et al., 2018**	Japan	< 70<br>≥ 70	< 70, n = 18<br>≥ 70, n= 23	Resection	83.3%, 42.8%, 30.6%	82.6%, 50.6%, 50.6%	p= 0.40
**Liu et al., 2014**	Taiwan	≥ 75<br>< 75	≥ 75, n= 129<br>< 75, n= 730	Resection	92%, 82%, 72%	93%, 82%, 61%	**p= 0.02****
**Faber et al., 2013**	Germany	< 70<br>≥ 70	< 70, n= 78<br>≥ 70, n= 63	Resection	78.5%, 56.5%, 47.1%	59.9%, 40.3%, 6.7%	**p< 0.001**
**Liu et al., 2014**	China	< 40<br>> 40	< 40, n= 70<br>> 40, n= 83	Resection	93%, 79%, 47%	85%, 75%, 40%	p= 0.11
**Portolani et al., 2011**	Italy	< 70<br>≥ 70	< 70, n= 276<br>≥ 70, n= 175	Resection	94.1%, 68.6%, 45.4%	83.7%, 62.7%, 31.4%	NS*
**Huang et al., 2009**	China	< 70<br>≥ 70	< 70, n= 268<br>≥ 70, n= 67	Resection	71.6%, 39.9%, 31.4%	83.3%, 54.6%, 43.2%	p= 0.16
**Jing-lin et al., 2008**	China	< 65<br>≥ 65	< 65, n= 776<br>≥ 65, n= 99	Resection	77.8%, 53.4%, 45.4%	84.6%, 65.3%, 59.5%	p= 0.12
**Zhou et al., 2006**	China	< 65<br>≥ 65	< 65, n= 125<br>≥ 65, n= 54	Resection	79.6%, 49.1%, 38.6%	88.8%, 56.8%, 50.1%	p= 0.38

**Ferrero et al., 2005**	Italy	< 70<br>≥ 70	≤ 70, n= 177<br>> 70, n= 64	Resection	74.1%, 49. 6%, 32.3%	81.3%, 57.1%, 48.6%	p= 0.08
**Yeh et al., 2004**	Taiwan	< 70<br>≥ 70	< 70, n= 398<br>≥ 70, n= 34	Resection	69%, 45.5%, 32.1%	85.3%, 64.3%, 39.6%	p= 0.32
**Hanazaki et al., 2000**	Japan	< 70<br>≥ 70	< 70, n= 283<br>≥ 70, n= 103	Resection	85.1%, 55.2%, 40.0%	83.4%, 51.0%, 42.2%	p= 0.66
**Cuchetti et al., 2015**	Italy	< 60<br>60 - 66<br>67 - 70<br>> 70	< 60, n= 229<br>60 – 66, n= 230<br>67- 70, n=231<br>> 70, n= 229	Resection	<60: 86%, 67%, 57%<br>60 - 66: 92%, 68%, 56%<br>67 - 70: 90%, 69%, 54%	>70: 89%, 68%, 44%	p= 0.40
**Mirici-Cappa et al., 2009**	Italy	< 70<br>≥ 70	< 70, n= 142<br>≥ 70, n= 43	Resection	86.5%, 61.6%, 32.4%	95.2%, 67.3%, 44.8%	p= 0.08
**Kaibori M et al., 2009**	Japan	< 70<br>≥ 70	< 70, n= 333<br>≥ 70, n= 155	Resection	*3-, 5-, 7-year OS:*<br>69.7%, 57.3%, 44.0%	*3-, 5-, 7-year OS:*<br>70.3%, 54.6%, 35.8%	p= 0.79
**Oishi et al., 2008**	Japan	< 75<br>≥ 75	< 75, n= 502<br>≥ 75, n= 64	Resection	*3-, 5-, 10-year OS:*<br>81%, 64%, 33%	*3-, 5-, 10-year OS:*<br>77%, 58%, 32%	NS*
**Wang W. et al., 2017**	China	< 70<br>≥ 70	< 70, n= 97<br>≥ 70, n= 48	Resection	*5-year OS*<br>55%	*5-year OS*<br>52%	p= 0.61
**Mercantini et al., 2002**	Italy	< 65<br>≥ 65	< 65, n= 32<br>≥ 65, n= 14	Resection	*5-year OS:*<br>41.8%	*5-year OS:*<br>77.4%	p= 0.30
**Yeh et al., 2002**	Taiwan	≤ 30<br>> 70	≤ 30, n= 32<br>> 70, n= 48	Resection	*1-, 5-year OS:*<br>52.2%, 30.4%	*1-, 5-year OS:*<br>84.4%, 42.1%	p= 0.08
**Mori et al., 2014**	Japan	< 70<br>≥ 70	< 70, n= 131<br>≥ 70, n= 64	Resection	*5-year OS:*<br>56.3%	*5-year OS:*<br>36.0%	**p= 0.01**
**Taniai et al., 2013**	Japan	≥ 75<br>< 75	≥ 75, n= 63<br>< 75, n= 353	Resection	*3-, 5- year OS:*<br>63.4%, 46.6%	*3-, 5- year OS:*<br>56.2%, 40.2%	p= 0.62
**Hirokawa et al., 2013**	Japan	< 70<br>≥ 70	< 70, n= 120<br>≥ 70, n= 100	Resection	93%, 79%, 64%	91%, 71%, 56%	p= 0.07
**Takeishi et al., 2011**	Japan	< 40<br>≥ 40	< 40, n= 13<br>≥ 40, n= 246	Resection	*5-, 10-year OS:*<br>84.6%, 55.9%	5-, 10-year OS:<br>59.2%, 30.7%	p= 0.06
**Okamura et al., 2018**	Japan	< 75<br>≥ 75	< 75, n= 310<br>≥ 75, n= 111	Resection	*3-year, 5-year OS:*<br>77.4%, 71.9%	3-year, 5-year OS:<br>77.7%, 59.0%	p= 0.31
**Inoue et al., 2018**	Japan	≥80<br><80	≥ 80, n= 45<br>< 80, n= 485	Resection	85.3%, 70.9%, 57.0%	87.8%, 81.0%, 72.9%	p= 0.48

**Tsujita 2 et al., 2012**	Japan	≥80<br><80	≥ 80, n= 23<br>< 80, n= 385	Resection	*3-year OS:*<br>84.8%	*3-year OS:*<br>95.7%	p= 0.56
**Nozawa et al., 2014**	Japan	< 70<br>70 – 80<br>≥ 80	< 70, n= 239<br>70 – 80, n = 172<br>≥ 80, n= 20	Resection	*5-year OS*<br>65.3%	*5-year OS*<br>70 - 80: 59.5%<br>≥ 80: 66.9%	p=0.44
**Ide et al., 2013**	Japan	≥ 75<br>< 75	≥ 75, n=64<br>< 75, n= 192	Resection	*5-year OS*<br>68%	*5-year OS*<br>58%	p=0.64
**Shimada et al., 2020**	Japan	≥ 80<br>65 - 79<br>< 65	≥ 80, n= 49<br>65 – 79, n= 363<br>< 65, n= 384	Resection	*5-year OS:*<br>< 65: 62%	*5-year OS:*<br>≥ 80: 62%<br>65- 79: 65%	p=0.86
**Tan et al., 2019**	Australi a	< 70<br>≥ 70	< 70, n= 239<br>≥ 70, n= 71	Resection	*5-year OS:*<br>68.5%	*5-year OS:*<br>62%	p=0.71
**Najjar et al., 2019**	USA	< 70<br>> 70	< 70, n= 588<br>> 70, n= 45	Liver transplant	*5-year OS:*<br>71%	*5-year OS:*<br>67%	p=0.35
**Felga et al., 2014**	Brazil	< 65<br>≥ 65	< 65, n= 141<br>≥ 65, n= 37	Liver transplant	81%, 70%, 66%	78%, 64%, 64%	p= 0.49
**Thomas et al., 2017**	USA	< 40<br>≥ 40	< 40, n= 320<br>≥ 41, n= 1,280	Liver transplant	*5-year OS*<br>74.4%	*5-year OS*<br>68.6%	p= 0.09
**Ahmed et al., 2021**	USA	< 65<br>≥ 65	< 65, n= 319<br>≥ 65, n= 116	Liver transplant	86.0%, 64.8%, 49.0%	82.5%, 59.0%, 44.8%	p= 0.13
**Liu et al., 2014**	Taiwan	≥ 75<br>< 75	≥ 75, n= 147<br>< 75, n= 337	Local ablation	95%, 81%, 72%	96%, 78%, 61%	p= 0.69
**Hiraoka et al., 2009**	Japan	< 75<br>≥ 75	< 75, n= 143<br>≥ 75, n= 63	Local ablation	93.2%, 78.3%, 57.5%	91.7%, 82.5%, 49.7%	p= 0.60
**Kao Wei-Yu et al., 2012**	Taiwan	≤ 65<br>> 65	≤ 65, n= 100<br>> 65, n= 158	Local ablation	98.0%, 87.0%,81.3%	93.9%, 72.4%, 65.4%	p= 0.01
**Zeng Jian et al., 2020**	China	≤ 40<br>> 40	≤ 40, n= 699<br>> 40, n= 3,805	Resection	81.2%, 61%, 45.3%	86.4%,65.3%, 47.7%	p= 0.01
**Fujiwara et al., 2014**	Japan	≥ 75<br>< 75	≥ 75, n= 353<br>< 75, n= 1,048	Local ablation	*Mortality Rate*<br>2.7%, 17.7%, 37.1%	*Mortality Rate*<br>4.5%, 24.4%, 47.3%	p< 0.001
**Pu et al., 2021**	China	≤ 35<br>> 70	≤ 35, n= 192<br>> 70, n= 339	Resection	80.2%, 62.2%, 49.1%	89%, 71.1%, 53.9%	p> 0.05
**Xing et al., 2020**	China	≥ 70<br>< 70	≥ 70, n= 259<br>< 70, n= 1,875	Resection	86.7%, 67.5%, 55.3%	89.2%, 69.6%, 49.4%	p= 0.03
**Kaibori et al., 2019**	Japan	40 - 59<br>60 - 74	40 - 59, n= 2,991	Resection	*5-year OS*<br>40 - 59: 68.8%	*5-year OS*<br>>75: 59.5%	p< 0.001

		> 75	60 - 74, n= 7,576<br>≥ 75, n= 2,020		60 - 74: 63.2%		
**Hsu et al., 2018**	Japan	< 70<br>≥ 70	< 70, n= 178<br>≥ 70, n= 79	Resection	79%, 57%, 51%	76%, 55%, 48%	p= 0.32
**Doi K et al., 2015**	Japan	< 70<br>≥ 70	< 70, n= 162<br>≥ 70, n= 118	Local ablation	*3-, 5-year OS*<br>70%, 52%	*3-, 5-year OS*<br>73%, 57%	p= 0.90
**Motoyama et al., 2015**	Japan	< 75<br>≥ 75	< 75, n= 499<br>≥ 75, n= 113	Resection	*5-year OS*<br>57.6%	*5-year OS*<br>46%	p= 0.02
**Hiroki Nishi et al., 2012**	Japan	< 75<br>≥ 75	< 75, n= 238<br>≥ 75, n= 130	Local ablation	97.6%, 83.7%, 64.0%	90.0%, 64.1%, 44.8%	p= 0.001
**Tan LY et al., 2021**	Singapore	< 70<br>70 - 79<br>≥ 80	< 70, n= 764<br>70 – 79, n= 278<br>≥ 80, n= 50	Resection	*1-, 5-, 10-years OS*<br><70: 91.5%, 62.1%, 46.0%	*1-, 5-, 10-years OS*<br>>80: 77.9%, 37.7%, 10.5%<br>70-79: 85.9%, 57.7%, 27.4%	<70 vs 70-79: p= 0.03<br><br>70-79 vs >80: p= 0.05
**Diao Y. et al., 2021**	China	< 40<br>≥ 40	< 40, n= 354<br>≥ 40, n= 1,684	Resection	83.2%, 67.2%, 57.7%	91.9%, 75%, 63.1%	p= 0.31
**Tsuchiya et al., 2020**	Japan	< 80<br>≥ 80	< 80, n= 68<br>≥ 80, n= 68	Local ablation	94.1%, 72.8%, 49.3%	98.5%, 87.9%, 50.5%	p= 0.83
**Chen Zheng et a., 2020**	China	< 65<br>≥ 65	< 65, n= 738<br>≥ 65, n= 92	Resection	68.9%, 42.0%, 33.2%	71.3%, 43.2%, 35.0%	p= 0.74
**Santambrogio et al., 2016**	Italy	< 75<br>≥ 75	< 75, n= 115<br>≥ 75, n= 53	Resection	*3-, 5-year OS*<br>82%, 60%	*3-, 5-year OS*<br>65%, 46%	p= 0.02
**Kishida N et al., 2015**	Japan	< 75<br>≥ 75	< 75, n= 82<br>≥ 75, n= 22	Resection	*5-year OS*<br>69%	*5-year OS*<br>81%	p= 0.35
**Nanashima et al., 2011**	Japan	< 50<br>50 - 69<br>70 - 79<br>≥ 80	< 50, n= 9<br>50 – 69, n= 110<br>70 – 79, n= 57<br>≥ 80, n= 12	Resection	*5-, 10-year OS*<br>< 50: 50%, 25%<br>50 - 69: 51%, 27%	*5-, 10-year OS*<br>70 - 79: 46%, 36%<br>> 80: 78%, N/A	p= 0.82
**Kim Josepth et al., 2013**	USA	35 - 49<br>50 - 64<br>≥ 65	35 - 49<br>50 - 64<br>≥ 65	Liver transplant	*1-, 5-year OS*<br>35 - 49: 89%, 67%<br>50 - 64: 87%, 65%	*1-, 5-year OS*<br>> 65: 85%, 60%	p= 0.001
**Schwartz et al., 2012**	USA	< 70<br>≥ 70	< 70, n= 22,296<br>≥ 70, n= 480	Liver transplant	*5-year OS*<br>72.7%	*5-year OS*<br>55.2%	N/A

**Cullaro et al., 2020**	USA	< 65<br>≥ 65	< 65, n= 22,607<br>≥ 65, n= 5,275	Liver transplant	5-year post-transplant mortality<br>20%	5-year post-transplant mortality<br>21%	p= 0.02

Abbreviations: OS, overall survival; NS, not significant.
*P-value not explicitly stated
**Not significant after study propensity score matching.

Table S3. Clinical Outcomes in Younger and Older Patients, Stratified by Study Period

Category	Younger Patients (%)	Older Patients (%)
**Curative Treatment Receipt**		
All studies	50.4 (40.0-60.6)	38.0 (27.6-48.4)
<2005	47.8 (9.9-85.7)	46.7 (4.6-88.8)
2005-2009	49.4 (14.1-84.6)	22.3 (6.0-38.6)
2010-2014	58.8 (39.7-77.9)	45.9 (22.8-69)
>2015	42.5 (18.4-66.6)	36.4 (12.3-60.6)
**Early-Stage HCC**		
All studies	36.3 (28.8-43.7)	35.0 (27.6-42.4)
<2005	29.1 (9.3-49.0)	32.6 (5.9- 59.2)
2005-2009	26.1 (4.3-47.8)	27.0 (4.9-49.1)
2010-2014	40.9 (28.6-53.2)	35.5 (26.0-45.1)
>2015	40.5 (29.5-51.4)	40.7 (26.7-54.7)

Table S4. Studies reporting differences in performance status, liver function, comorbidities, and curative treatment receipt in younger versus older patients.

Study, Year	Country	Age Cutoff	Performance Status*	Liver Function**	Comorbidities[γ]	Curative Treatment Receipt
**Liu et al., 2014**	Taiwan	< 75<br>≥ 75	Older < Younger	No difference	N/A	Older < Younger
**Yan H. et al., 2022**	China	≤ 45<br>> 45	N/A	Older < Younger	N/A	Older < Younger
**Seo et al., 2019**	South Korea	75-80<br>80-85<br>≥ 85	No difference	No difference	No difference	Older < Younger
**Oweira et al., 2017**	USA	< 70<br>≥ 70	N/A	N/A	N/A	Older < Younger
**Borzio et al., 2016**	Italy	≤ 70<br>> 70	Older < Younger	Older < Younger	Older > Younger	Older < Younger
**Yan M. et al., 2017**	USA	Varied	N/A	N/A	N/A	Older < Younger
**Lee Sang H et al., 2011**	South Korea	< 65<br>≥ 65	Older < Younger	No difference	Older > Younger	Older < Younger
**Tseng Po-Lin et al., 2010**	China	< 70<br>≥ 70	N/A	N/A	N/A	Older < Younger
**Mirici-Cappa et al., 2009**	Italy	< 70<br>≥ 70	N/A	Older > Younger	Older > Younger	Older < Younger
**Chang et al., 2008**	Singapore	≤ 40<br>> 40	N/A	N/A	N/A	Older < Younger
**Pignata et al., 2006**	Italy	< 70<br>≥ 70	N/A	No difference	N/A	Older < Younger
**Tsukioka et al., 2006**	Japan	< 80<br>≥ 80	Older < Younger	No difference	N/A	Older < Younger
**El-Serag et al., 2006**	USA	Varied	N/A	N/A	No difference	Older < Younger
**Dohmen et al., 2004**	Japan	< 80<br>≥ 80	N/A	No difference	N/A	No difference
**Lam et al., 2004**	China	≤ 40<br>> 40	N/A	Older < Younger	Older > Younger	No difference

**Giannini et al., 2002**	Italy	< 65<br>≥ 65	N/A	No difference	N/A	No difference
**Guo et al., 2017**	China	< 65<br>≥ 65	Older < Younger	Older < Younger	Older > Younger	Older < Younger
**Fan et al., 2013**	China	< 70<br>≥ 70	Older < Younger	No difference	Older > Younger	Older < Younger
**Kim et al., 2012**	South Korea	< 70<br>≥ 70	No difference	No difference	Older > Younger	Older < Younger
**Kozyreva et al., 2011**	USA	< 70<br>≥ 70	Older < Younger	No difference	Older > Younger	Older < Younger
**Ratana-Amornpin et al., 2021**	Thailand	< 65<br>≥ 65	N/A	No difference	Older > Younger	No difference
**Ozenne et al., 2011**	France	< 75<br>≥ 75	No difference	No difference	N/A	Older < Younger
**Hori et al., 2014**	Japan	< 75<br>≥ 75	N/A	N/A	N/A	Older < Younger

Abbreviations: N/A, not applicable.
* Performance status using Eastern Cooperative Oncology Group (ECOG) when available.
** Liver function using Child-Pugh score when available.
[γ] Reported comorbidities may vary depending on study and available data, though typically included diabetes and hypertension.

Table S5. Studies reporting absolute overall survival estimates for younger and older patients

Author, Year	Type of treatment receipt	Number of Younger Patients	Number of Older Patients	Age cut-off used	Survival
**Fujiwara N. et al, 2014**	Local ablation	1,048	353	75 years	**1-, 3-, 5-year Mortality:**<br>Older: 4.5%, 24.4%, 47.3%<br>Young: 2.7%, 17.7%, 37.1%
**Lee et al, 2022**	Not specified	4,294	2,147	65 years	**1-, 3-, 5-year OS:**<br>Older: 59%, 45.5%, 33.5%<br>Young: 60%, 46.3%, 35.7%
**Pu et al, 2022**	Surgical resection	192	339	>70 vs. <35 years	**1-, 3-, 5-year OS:**<br>Older: 89%, 71.1%, 53.9%<br>Young: 80.2%, 62.2%, 49.1%
**Kaibori et al, 2019**	Surgical resection	10,567	2,020	75 years	**5-year OS:**<br>40 - 59 yr: 68.8%<br>60 - 74 yr: 63.2%<br>>75: 59.5%
**Seo et al., 2019**	Surgical resection	346	204	80 years	**Median OS:**<br>Older: 38.5 +/- 8.3 months<br>Young: 45.8 +/- 2.6 months
**Motoyama et a., 2014**	Surgical resection	499	113	75 years	**5-year OS:**<br>Older: 57.6%

					Young: 46%
**Hiroki Nishi et al., 2012**	Local ablation	238	130	75 years	**The 1-, 3- and 5-year OS**<br>Older: 90.0%, 64.1% and 44.8%<br>Young: 97.6%, 83.7% and 64.0%
**Kao Wei-Yu et al., 2012**	Local ablation	100	158	65 years	**1, 3, and 5-year OS**<br>Older: 93.9%, 72.4%, and 65.4%<br>Younger: 98.0%, 87.0%, and 81.3%
**Lee Sang et al., 2011**	Combined treatments	149	113	65 years	**1- and 3-year OS**<br>Older: 79.5% and 30.4%<br>Young: 68.7% and 39.5%
**Tseng et al., 2010**	Combined treatments	55	33	70 years	**4-year OS**<br>Young: 57.4%<br>Older: 28.9%
**Chang et al., 2008**	Combined treatments	55	583	40 years	**Median OS**<br>Young: 51.2 months<br>Older: 11.6 months
**Pignata et al., 2006**	Combined treatments	158	492	70 years	**1-year OS**<br>Older: 53.5%<br>Young: 60.2%
**Tan et al., 2021**	Surgical resection	764	328	70 years	**1-, 5-, 10-years OS**<br>>80: 77.9%, 37.7%, 10.5%

					70-79: 85.9%, 57.7%, 27.4%<br><70: 91.5%, 62.1%, 46.0%
**Diao et al., 2021**	Surgical resection	354	1,684	40 years	**1-, 3-, 5-year OS:**<br>Older: 91.9%, 75%, 63.1%<br>Young: 83.2%, 67.2%, 57.7%
**Tsuchiya K et al., 2020**	Local ablation	68	68	80 years	**1-, 3-, 5-year OS:**<br>Older: 98.5%, 87.9%, and 50.5%<br>Young: 94.1%, 72.8%, and 49.3%
**Xing Hao et al., 2019**	Surgical resection	1,875	259	70 years	**1-, 3-, 5-year OS:**<br>Older: 89.2%, 69.6%, 49.4%<br>Young: 86.7%, 67.5%, 55.3%
**Chen Zheng et al., 2020**	Surgical resection	738	92	65 years	**1-, 3-, 5-year OS:**<br>Older: 71.3%, 43.2%, 35.0%<br>Young: 68.9%, 42.0%, 33.2%
**Li Lian et al., 2019**	Surgical resection	84	84	60 years	**1-year and 5-year OS:**<br>Older: 73.9% and 37.3%<br>Young: 54.2% and 22.2%
**Hsu et al., 2018**	Surgical resection	178	79	70 years	**1-, 3-, 5-year OS:**<br>Older: 76%, 55%, and 48%<br>Young: 79%, 57%, and 51%
**Borzio et al., 2016**	Combined treatments	527	542	70 years	**Median OS:**

					Older: 29 months<br>Young: 33 months
**Guo Hui et al., 2017**	Combined treatments	1,212	318	65 years	**Median OS**<br>Older: 27 months<br>Young: 33 months
**Santambrogio et al., 2016**	Surgical resection	115	53	75 years	**3-, 5-year OS:**<br>Older: 65%, 46%<br>Young: 82%, 60%
**Kishida N et al., 2016**	Surgical resection	82	22	75 years	**5-year OS:**<br>Older: 81%<br>Young: 69%
**Doi K et al., 2015**	Local ablation	162	118	70 years	**3- and 5-year OS:**<br>Older: 73% and 57%<br>Young: 70% and 52%
**Fan et al., 2014**	Combined treatment	531	164	70 years	**Median OS:**<br>Older: 26.2 months<br>Young: 28.3 months
**Nanashima et al., 2011**	Surgical resection	119	69	70 years	**5-, 10-year OS:**<br>< 50: 50%, 25%<br>50 - 69: 51%, 27%<br>70 - 79: 46%, 36%<br>> 80: 78%

**Yau Thomas et al., 2009**	TACE	2,452	550	70 years	**1-, 3-, 5- year OS:**<br>Older: 54.4%, 23.2%, 10.6%<br>Young: 39.2% 14.9% 8.4%
**Kim Joseph et al., 2013**	Liver transplant	8,625	1,613	65 years	**1-, 5-year OS:**<br>35 - 49: 89%, 67%<br>50 - 64: 87%, 65%<br>> 65: 85%, 60%
**Xiao Jun et al., 2014**	TACE	1,877	616	60 years	**1- and 3-year OS**<br>Young: 31.8% and 6.5%<br>Older: 84.7% and 28.9%
**Roth G et al, 2022**	TACE	183	88	70 years	**Median OS**:<br>Older: 31.0 months<br>Young: 45.1 months
**Cullaro et al., 2020**	Liver transplant	22,607	5,275	65 years	**1-, 5- year post-OLT Mortality:**<br>Elderly: 10%, 21%<br>Young: 8%, 20%
**Schwartz et al., 2012**	Liver transplant	22,296	480	70 years	**1, 3, 5- year OS:**<br>Older: 83.0%, 67.3%, and 54.5%<br>Young: 89.4%, 76.7%, 67.8%
**Cohen M et al., 2013**	TACE	79	23	75 years	**1, 2, 3-year OS**<br><65: 74%, 37%, 31%<br>65-75: 83%, 66%, 48%

					≥75: 86%, 41%, 23%
**Hajiev et al., 2021**	Sorafenib	4,806	792	75 years	**Median OS**<br>Older: 7.3 (95% CI: 7.0–7.6) months<br>Young: 7.2 (95% CI: 6.4–8.0) months
**Wong Hilda et al., 2011**	Sorafenib	137	35	70 years	**Median OS (months)**<br>Older: 5.32 (4.44–10.35) months<br>Young: 5.16 (4.21–6.05) months
**Nishikawa H et al., 2014**	Sorafenib	279	179	75 years	**Median OS**<br>Older: 9.7 (95% CI, 7.5-12.0) months<br>Young: 8.2 (95% CI, 6.9-9.6) months

Abbreviations: OS, overall survival; TACE, transarterial chemoembolization

Table S6. Studies reporting liver vs. non-liver mortality for younger and older patients

Author, Year	Type of treatment receipt	Number of younger patient liver related deaths	Number of older patient liver-related deaths	Number of younger patient non-liver related deaths	Number of older patient non-liver related deaths
Lee et al., 2011	Any treatment	68/149	62/113	4/149	2/113
Kozyreva et al., 2011	Any treatment	140/240	46/95	20/240	21/95
Adeniji et al., 2020	Liver transplant	12/4001	11/979	20/4001	18/979
Kim et al., 2013	Liver transplant	875/8625	149/1613	1536/8625	377/1613
Pu et al, 2022	Surgical resection	11/192	93/339	100/192	96/339
Motoyama et a., 2014	Surgical resection	224/499	41/113	26/499	15/113
Santambrogio et al., 2016	Surgical resection	39/115	19/53	13/115	11/53
Fujiwara. et al, 2014	Local ablation			81/1048	52/353
Hiroki Nishi et al., 2012	Local ablation	51/238	27/130	7/238	13/130
Kao et al., 2012	Local ablation	8/100	31/158	2/100	4/158
Tsuchiya et al., 2020	Local ablation	16/68	17/68	11/68	7/68
Masconi et al., 2020	Chemoembolization	66/145	43/80	15/145	16/80
Yau et al., 2009	Chemoembolization	691/2452	145/550	29/2452	29/550

Table S7. Studies comparing outcomes between younger and older patients with early-stage HCC

Author, Year	Staging System	Type of treatment receipt	Age cut-off	Proportion of younger patients undergoing curative treatment	Proportion of older patients undergoing curative treatment
**Oweira et al, 2017**	TNM I & II	Surgical Resection, liver transplantation, local ablation	70 – 80 vs. > 80 years	1,876/4,563	581/2,130
**Yan H et al, 2022**	BCLC 0-B	Surgical Resection	45 years	12/207	43/1395
**Tseng et al, 2010**	BCLC 0-A	Surgical Resection, local alation	70 years	19/55	7/33*
**Yan M et al, 2017**	Milan	Surgical Resection, liver transplantation	50 years	Resection: 565/4,697<br>Transplant: 392/4,697	Resection: 3,740/37,791<br>Transplant: 2,119/37,791
**Guo H et al, 2017**	BCLC 0-A	Local ablation	65 years	86/1,212	32/318
**Tsukioka G et al, 2006**	TNM I & II	Liver transplantation, local ablation	50 – 60 vs. >80 years	N/R	N/R
**Lee et al, 2011**	BCLC 0-A	Surgical Resection, liver transplantation, local ablation	65 years	N/R	N/R
**Borzio et al, 2016**	BCLC 0-A	Surgical Resection, liver transplantation, local ablation	70 years	N/R	N/R
**Kim Yun et al, 2012**	BCLC 0-A	Surgical Resection, liver transplantation, local ablation	70 years	N/R	N/A
